# ORDerly: Data Sets and Benchmarks for Chemical Reaction Data

**DOI:** 10.1021/acs.jcim.4c00292

**Published:** 2024-04-22

**Authors:** Daniel S. Wigh, Joe Arrowsmith, Alexander Pomberger, Kobi C. Felton, Alexei A. Lapkin

**Affiliations:** Department of Chemical Engineering and Biotechnology, University of Cambridge, Cambridge CB3 0AS, U.K.

## Abstract

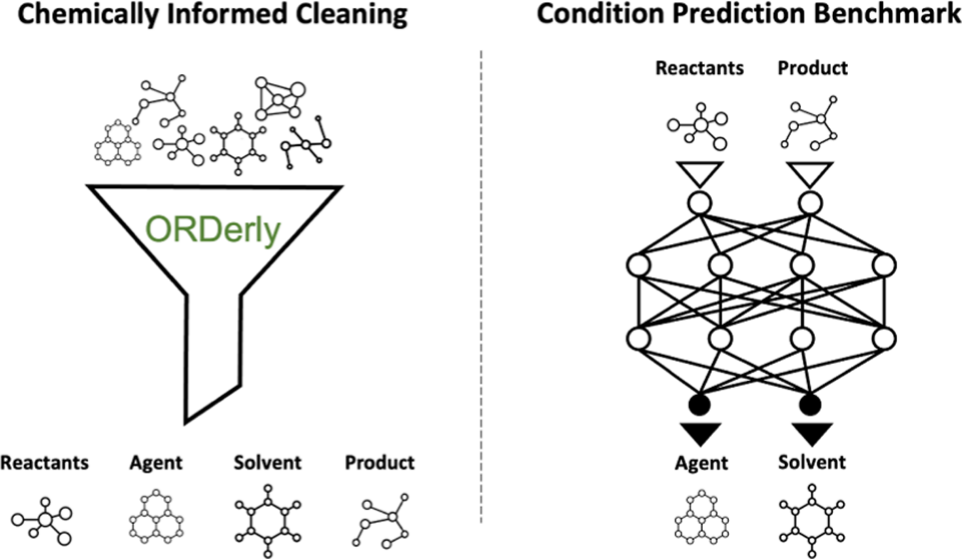

Machine learning has the potential to provide tremendous value to life sciences by providing models that aid in the discovery of new molecules and reduce the time for new products to come to market. Chemical reactions play a significant role in these fields, but there is a lack of high-quality open-source chemical reaction data sets for training machine learning models. Herein, we present ORDerly, an open-source Python package for the customizable and reproducible preparation of reaction data stored in accordance with the increasingly popular Open Reaction Database (ORD) schema. We use ORDerly to clean United States patent data stored in ORD and generate data sets for forward prediction, retrosynthesis, as well as the first benchmark for reaction condition prediction. We train neural networks on data sets generated with ORDerly for condition prediction and show that data sets missing key cleaning steps can lead to silently overinflated performance metrics. Additionally, we train transformers for forward and retrosynthesis prediction and demonstrate how non-patent data can be used to evaluate model generalization. By providing a customizable open-source solution for cleaning and preparing large chemical reaction data, ORDerly is poised to push forward the boundaries of machine learning applications in chemistry.

## Introduction

Advancements in chemistry and materials science hinge on the availability of high-quality chemical reaction data, and the advent of machine learning (ML) for science has highlighted the value that data can bring to chemistry. One important application is in the pharmaceutical industry, where figuring out how to make novel molecules remains a significant bottleneck, causing delays in the “make” step of the “design, make, test” cycle.^1^ Making a molecule (product) includes predicting the reaction pathway (retrosynthesis) and suitable reaction conditions (e.g., solvents and reagents) and optimizing for one or more outcomes such as reaction yield, selectivity, and conversion. ML is well suited to assist with these tasks, with a range of tools being developed for forward reaction prediction,^2−4^ retrosynthesis,^5−10^ condition prediction,^11,12^ yield prediction,^13−15^ and closed-loop optimization.^16−18^ A more formal definition of these reaction-related tasks can be found in the Supporting Information.

Building reaction prediction tools requires access to large data sets for training. Historically, researchers have accessed proprietary in-house data sets or acquired the data through commercial data services such as Reaxys^19,20^ and SciFinder.^21^ The advantage of commercial databases is both the scale of the data sets available (often millions of reactions) and the annotation already completed by the publishers. Yet, these data sets are not freely available to ML practitioners, stymieing advances in reaction condition prediction in both academia and industry. Recently, efforts have been made to create openly accessible databases for chemical reaction data. In particular, the Open Reaction Database (ORD)^22^ is promising due to its exhaustive schema for describing chemical reaction data and breadth of data already incorporated. Yet, many of the data sets in ORD require further processing before they can be used in ML pipelines, preventing practical use. This is especially true for the largest data set in ORD extracted from the United States (US) patent literature (the “USPTO data set”^23^). In this work, we endeavor to close this gap.

Herein, we present ORDerly, a new framework for extracting and cleaning data from ORD, accompanied by data sets for three reaction-related tasks: retrosynthesis, forward, and condition prediction. By offering an open-source and customizable solution for cleaning chemical reaction data, ORDerly aims to contribute to the development of advanced ML models in chemistry and material science.

### Chemical Reaction Cleaning Tools

Most existing tools for cleaning reaction data is primarily targeted at retrosynthesis and forward prediction tasks^24−27^ and have somewhat limited extensibility, given that they are built to take as inputs CSV files or the stationary XML files of the USPTO data set^23^ instead of the outputs of continuously updated databases such as ORD.^22^ Furthermore, in these publications, there is little to no discussion of how decisions made during cleaning (e.g., restricting the number of components in a reaction or the minimum frequency of occurrence) impact the data sets being cleaned or performance of models trained on the data sets. Gimadiev et al.^29^ presented a 4-step protocol for cleaning of molecular structures using data originating from Reaxys, USPTO, and Pistachio^28^ (e.g., functional group standardization, valence checking) as well as curation of the reaction transformation (e.g., via reaction balancing or atom mapping), but no further application such as predictive modeling was conducted. Andronov et al. published a cleaning pipeline involving atom-mapping, removal of isotope information, and SMILES canonicalization for subsequent training of a transformer model for single-step retrosynthesis.^30^ ORDerly took inspiration from these previously published works to develop an open-source cleaning pipeline integrated with ORD, providing numerous reaction task benchmarks that have undergone in silico validation.

USPTO, being the largest open-source chemical reaction data set, has been cleaned a number of times for different learning tasks. For example, the USPTO-50K^31,32^ and USPTO-MIT data sets^33^ are commonly used for benchmarking single-step retrosynthesis and forward prediction models,^a^ and these benchmarks are available in aggregate benchmarking sets such as the Therapeutics Data Commons (TDC).^34^ However, the code used to process the raw data to generate the aforementioned USPTO benchmarks was not published, and there is no publicly available benchmark for reaction condition prediction extracted from these data sets. Even though the data in ORD is stored in accordance with a structured schema, we found that further effort is required to transform the labeled data into ML-ready data sets.

### Forward Prediction and Single-Step Retrosynthesis Models

Forward prediction and single-step retrosynthesis models both need to predict how bonds might be broken and formed to produce new molecules. A common approach is to enumerate a set of templates for bond changes that happen in particular classes of reactions and use a classifier to predict the most likely template given a set of molecules.^3,35−39^ Alternatively, some models have been designed to explicitly predict bond changes.^33,40^ One promising approach is to directly predict the SMILES strings of the reactants (single-step retrosynthesis) or products (forward prediction) using a natural language processing model such as a transformer.^2,6,7,10^ In this work we use the transformer architecture of Schwaller et al.^2^

### Condition Prediction Models

Numerous approaches to predicting suitable reaction conditions have been proposed over the years. Struebing et al. used quantitative structure–activity-relationship (QSAR) to identify the most suitable solvents.^41^ Several later approaches focused on indirect prediction of conditions by learning to predict a measure of reaction performance, such as yield, and then subsequently ranking and recommending conditions.^42−44^ Using a different strategy, Kwon et al.^45^ and Schwaller et al.^10^ relied on generative modeling approaches to predict reaction conditions, and Walker et al. used network analysis^46^ to cluster chemical reactions, using the insight that similar reactions often require similar conditions (particularly in the case of solvents), thus mimicking how chemists reason about chemical reaction conditions. Afonina et al. applied a likelihood ranking model delivering a list of conditions ranked according to their suitability.^47^ While good performance was achieved, the approach was limited in scope, focusing on only hydrogenation reactions. Gao et al.^11^ built a model for reaction condition prediction agnostic of reaction class for sequential prediction of catalyst, solvents, agents, and temperature using approximately ten million reactions mined from a closed-source data set, Reaxys.^19,20^ We train this model with minor modifications on our new open-source condition prediction benchmark.

## Methodology

ORDerly uses cleaning operations motivated by a first-principles understanding of chemistry and is split into an extraction script and a cleaning script. This enables users to extract the data they desire and more easily clean it in different ways for different applications.

### Extraction

#### Specification of Data Source

Users can choose whether all data in ORD should be extracted, or only a subset (e.g., all of USPTO, everything except USPTO). This enables users to, for example, train models with data from one source and test their performance with data from another source. Creating test sets from different data sources is a robust way to evaluate the generalization performance. The following items are extracted from each reaction: the mapped reaction string; the labeled reactants, products, catalysts, and agents; the temperature; the yield(s); and the procedure details.

#### Canonicalization and Conversion of Molecule Names

Canonicalization of molecular SMILES and names is an important step in any cleaning pipeline to ensure that the same molecule is always referred to in the same way; particularly when using one-hot encoding (OHE). A CSV file is created to keep track of all non-SMILES names used to represent molecules and to keep track of frequently used molecule names. We then manually built a name resolution dictionary to replace the molecular names with the corresponding SMILES strings. We also added mappings for different representations of the same catalyst to ensure canonical representation. As an example, tetrakistriphenylphosphine palladium/Pd(Ph3)4/Pd[PPh3]4 appeared with many different names and even with different SMILES strings (different numbers of ligands in the SMILES strings); these were canonicalized using the name resolution dictionary we built. Researchers are welcome to download this dictionary from the ORDerly GitHub repository and use it for their own projects.

#### Canonicalization of SMILES

All SMILES strings are sanitized and canonicalized by the cheminformatics package RDKit.^48^

#### Reaction Role Assignment

The extraction script allows the user to choose whether reaction roles should be assigned using the labeling in ORD (referred to as “labeling”) or using chemically informed reaction logic on the atom-mapped reaction string (referred to as “rxn string” or “reaction string”). Our reaction logic identified reactants [molecules that contribute heavy (non-hydrogen) atoms to the product(s)] and spectator molecules [molecules that do not contribute heavy atoms to the product(s)] based on the atom mapping and their position in the reaction SMILES string. An exception was added for hydrogen molecules, allowing hydrogen molecules to be labeled as reactants (e.g., in hydrogenation reactions) despite not contributing a heavy atom. Contribution of a hydrogen atom from a hydrogen molecule can be difficult to detect since hydrogen atoms are usually implicit in SMILES strings. Solvents were identified in the list of spectator molecules by cross checking against a list of solvents we compiled from prior research (see the Supporting Information), while all other spectator molecules were marked as agents.

### Cleaning

#### Remove Reactions without any Reactants or Products

Reactions without reactants and products do not make sense; therefore, these were removed.

#### Remove Reactions with too Many Components

Users are able to set the maximum number of each component in a reaction (e.g., delete any reactions with two or more products). The available components to choose from in the reaction string data sets are reactants, products, solvents, and agents. Only keeping reactions with one product can help to filter out multistep reactions, and setting a limit on the number of solvents can ensure compatibility with ML models that expect a certain number of components. Note that binary salts are usually represented with charge and separated by “.” (e.g., “[Na+].[Cl–]”), and thus count as two components.

#### Ensuring Consistent Yield

We added functionality to sanitize the yields of a reaction, i.e. checking that each individual yield as well as the sum of all yields is between 0 and 100%. However, since yield data is known to be much more noisy than structure data, this functionality is switched off by default and should be switched off for structure-related tasks (e.g., reaction condition prediction).

#### Frequency Filtering

**Figure 1:**
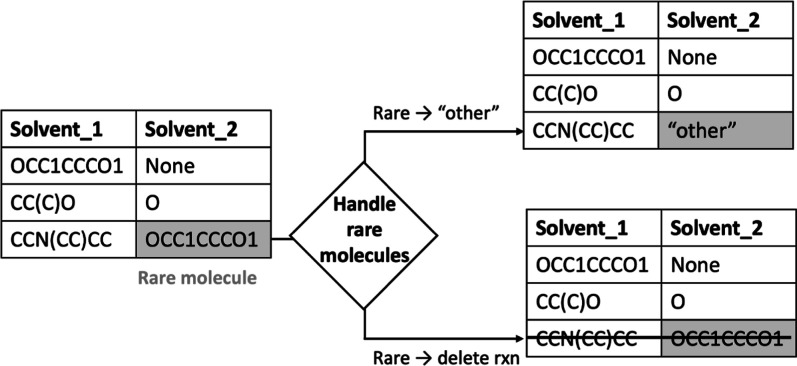
We present two different approaches for handling rare molecules. Rare → “other” is investigated as a strategy to avoid deleting reactions with rare molecules. When a rare solvent or agent is encountered with the Rare → delte rxn strategy, the full reaction is deleted.

Removing rare molecules can increase the signal-to-noise ratio in a data set by removing outliers and potentially erroneous reactions/molecules. Chemical reaction data is notoriously noisy, and this is particularly true of data from patents. Using reaction conditions that worked for others is a common strategy in chemistry, so when encountering reaction conditions (e.g., a reagent molecule) never (or exceedingly rarely) seen before in the data set of 1.7 million reactions, it is possible that the conditions were actually a mistranscription, thus motivating the removal of these rare occurrences. In this work, we investigated two different strategies for filtering spectator molecules based on their frequency: deleting the whole reaction if a rare spectator molecule is identified (rare → delete rxn), or keeping the reaction but mapping the rare molecules to an “other” category (rare → “other”) (see Figure 1). We conducted experiments with both the rare → delete rxn and rare → “other” strategies for the task of condition prediction. The frequency threshold was set at 100 in line with previous research,^11^ though the sensitivity of data set size to frequency threshold was still investigated (see the Supporting Information). Deleting reactions with rare molecules may create a more cohesive data set by removing outliers while renaming rare molecules “other” allows more reactions to be kept, offering more training data for the model. Note that we have also made available a data set for condition prediction without rare solvents and agents removed (see the Supporting Information).

#### Drop Duplicates

Duplicate reactions are removed.

#### Apply Random Split

The final step in the cleaning pipeline is to apply a random split to create training/test sets, carefully ensuring that any inputs present in the train set (i.e., reactants and products for reaction condition prediction) are not also present in the test set.

### Computational Details

All extraction/cleaning operations described in this section were performed using a 2022 Mac Studio with an Apple M1Max chip and 32GB of memory. In ORD there are roughly 1.7 million reactions from US patents (USPTO) and 94,000 reactions that are not from US patents. During handling of the USPTO data in ORD, we found that extracting and sanitizing the reaction components using the ORD labeling of components was slightly faster than using our custom logic applied to the reaction string, taking 28 and 48 min, respectively. The cleaning steps took 6–8 min. Due to the amount of non-patent data being much less, extraction and cleaning of non-USPTO data took only a few minutes.

### Data set Composition

Data sets generated with ORDerly have the following column groups:

- Reaction SMILES (string), is_mapped (bool)
- Reactants & products (SMILES strings)
- Solvents and agents (rxn string data), or solvents, catalysts, and reagents (labeling data) (SMILES strings)
- Temperature (Celsius), reaction time (hours), yield (%) (floats)
- Procedure details (string)
- Grant date (datetime), date of experiment (datetime), file name (string)

We used ORDerly to create benchmark data sets for three tasks: forward, retrosynthesis, and condition prediction using USPTO (atom-mapping: Indigo^49^). Several different data sets were created for each task, and the impact of each cleaning step on the data set size can be found in Table 1. The data sets are freely available and can be downloaded immediately from FigShare or regenerated using the code in the ORDerly Github repository (see the Data Availability Statement section for links).

**Table 1:** Number of Reactions Left in Each Dataset after Cleaning^a^

data set name	ORDerly-condition (labeling)	ORDerly-condition (rxn string)	ORDerly-forward	ORDerly-retro	non-USPTO-forward
full data set	1,771,032	1,771,032	1,771,032	1,771,032	94,043
too many reactants	518,369	1,627,929	1,743,179	1,627,929	46,821
too many products	473,437	1,589,977	1,740,254	1,589,977	43,362
too many solvents	446,484	1,385,579	1,689,075	NA	39,114
too many agents	446,484	1,279,207	1,552,671	NA	32,243
no reactants/products	441,859	1,261,701	1,533,571	1,564,525	32,103
dropping duplicates	264,846	753,338	919,077	939,648	29,417
frequency filtering	258,273	691,142	NA	NA	NA

a A description of each data set can be found in the Methodology section. Note that the actual number of reactions used for training will differ from the data set size shown below due to train/test splits and augmentation. Non-USPTO-retro had a final data set size of 23,334 and was cleaned in the same way as ORDerly-retro.

### Forward Prediction Benchmark

ORDerly-forward is a benchmark created from USPTO data in ORD for forward prediction consisting of reactions with up to two products and three reactants, solvents, and agents. A random 80/10/10 train/val/test split was applied to the benchmark. An additional test set called non-USPTO-forward was created by using all non-USPTO data in ORD (as of February 20, 2024) and cleaning it with the same parameters as those used for ORDerly-forward. No frequency filtering was applied.

### Single-Step Retrosynthesis Benchmark

ORDerly-retro is a benchmark created from USPTO data in ORD for retrosynthesis prediction consisting of reactions with one product and up to two reactants. A random 80/10/10 train/val/test split was applied to the benchmark. An additional test set called non-USPTO-retro was created by using all non-USPTO data in ORD (as of February 20, 2024) and cleaning it with the same parameters as those used for ORDerly-retro. No frequency filtering was applied.

### Condition Prediction Benchmark

ORDerly-condition is a benchmark data set created from USPTO data in ORD for reaction condition prediction and is, to the best of our knowledge, the first open-source reaction condition benchmark. Each reaction in ORDerly-condition contains one product and up to two reactants, two solvents, and three agents. A minimum frequency of 100 for the spectator molecules was applied.

## Results and Discussion

Experimental evaluation of the ORDerly-forward and ORDerly-retro benchmarks was performed using the Molecular Transformer architecture built by Schwaller et al.^2,50,51^ To switch from forward prediction to retrosynthesis prediction no changes to the transformer architecture were necessary, only the data was changed. The ORDerly-condition benchmark was evaluated together with the impact of different approaches to reaction role assignment and frequency filtering using the neural network architecture built by Gao et al.^11^

### Forward and Retrosynthesis Prediction with Transformers

Transformers were applied to two tasks: forward prediction (predicting products given reactants, solvents, and agents) and retrosynthesis (predicting reactants given a product). For the task of forward reaction prediction two different modes were tested: mixing the reactants, solvents, and agents in the SMILES string, or weakly separating the reactants from the solvents and agents with a ”>” token. Untokenized examples of transformer model inputs are shown in (1–3). Forward prediction with mixed inputs is a more difficult task since it is less obvious which atoms (characters) will appear in the product.
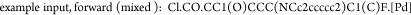
1
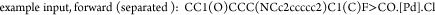
2
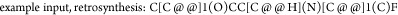
3

For both forward and retrosynthesis prediction, the order of the molecules was randomized, and the data set was augmented by replacing each SMILES string in the reaction with a random equivalent SMILES string (thus doubling the data set size), before finally being tokenized.^2^ Performance metrics are reported in Table 2, showing that across all tasks, only a small percentage of the generated SMILES strings are invalid.

**Table 2:** Test Performance with Molecular Transformer on Forward Prediction and Retrosynthesis (%)^a^

test sets	random split from USPTO			non USPTO		
tasks	invalid SMILES	accuracy (with SC)	accuracy (w/o SC)	invalid SMILES	accuracy (with SC)	accuracy (w/o SC)
forward (separated)	0.34	83.86	85.84	0.40	66.10	66.92
forward (mixed)	0.36	81.96	83.99	0.27	84.12	85.20
retrosynthesis	0.21	51.28	52.30	0.27	37.22	37.42

a The first column shows the percentage of invalid SMILES strings produced by the transformer (lower is better), while the second and third column show the top-1 accuracy with and without consideration of stereochemistry (SC), respectively (higher is better). Accuracy with non-USPTO test data for the task of retrosynthesis and forward (separated) is markedly lower than when using USPTO data, which is due to failure of reactant/agent separation.

Using a random split from USPTO as test set, the accuracies achieved on the forward prediction tasks are similar (albeit slightly lower) to the accuracies reported by Schwaller et al.^2^ (88–90% top-1 accuracy when trained on the USPTO_MIT^33^ data set), though the accuracies are not directly comparable since different subsets of USPTO were used. As expected, the performance with separated agents is higher than that with mixed agents, since it is an easier task, and it is encouraging to see that the models accurately predict stereochemistry. Accuracy with the retrosynthesis model on the held-out test set was roughly 50%, which is similar to previous work on retrosynthesis.^36^

Model accuracy on the non-USPTO test sets varied significantly by task. For forward (mixed), the accuracy achieved was similar between the USPTO and non-USPTO test sets, while for the forward (separated) and retrosynthesis tasks, the accuracy was significantly worse. This observation can be explained when considering the fact that from the 20,000+ reactions in the non-USPTO test sets, none of them contain a reaction string. ORDerly was therefore forced to rely on the ORD labeling to build the data set, which routinely mislabels agents as reactants. Consider as an example the following reaction found in the non-USPTO test set:
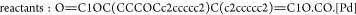
4
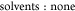
5
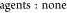
6
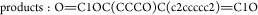
7

This reaction will confuse the retrosynthesis model since it has only been trained to predict reactants (molecules that contributes atoms to the product); it will never have had to predict a palladium atom during training. The forward (separated) model will be similarly confused, since it has been trained in the context of all molecules before the first ”>” being reactants, and all molecules after the first ”>” being agents, but that would not be the case for this reaction. In contrast, for forward (mixed) the reactants, agents, and solvents were mixed together during training, so the mislabeling of this reaction would not impact predictive accuracy. This mislabeling of agents was also encountered when building condition prediction data sets using the ORD labeling.

While it would, in principle, be possible to build reaction strings and map them, ORDerly was built to strictly operate downstream of ORD, and updating or otherwise changing the data in ORD is an upstream task. Furthermore, atom mapping is a computationally expensive task and would take away from ORDerly being a lightweight program to quickly generate ML data sets.

### Computational Details

The transformer models were trained for around 70 h (roughly 1000 epochs) on a Tesla T4 cloud GPU instance provided by lightning.ai. Evaluation was done with a model that was constructed by averaging the final 20 checkpoints.

### Reaction Condition Prediction with Neural Networks

The reaction condition prediction model used in this work predicts five categorical variables: two solvents and three agents. These five molecules form a set (order invariant), though the loss function in the model used to predict the molecules considers them sequentially (with order) since this was found to work better in practice.^11^ The metric used to evaluate the accuracy of the model should be order invariant, since the problem is order invariant, and for this reason, the accuracy metric used is top-3 exact match combination accuracy for each type of component (i.e., solvent, agent) and also for all components together (see Table 3). Beam search was used to identify the top-3 highest probability sets of reaction conditions. The top-3 accuracy was compared to the baseline predictive accuracy of simply predicting on the test set the most common molecules found in the train set.

**Table 3:** Top-3 Metrics on Condition Prediction with the Model Architecture of Gao et al.:^11^ Frequency Informed Guess Accuracy//Model Prediction Accuracy//AIB %

data sets	labeling	labeling	reaction string	reaction string
	rare → “other”	rare → delete rxn	rare → “other”	rare → delete rxn
solvents	57//70//31%	58//71//31%	36//51//24%	35//50//23%
agents	91//94//26%	92//94//23%	46//56//20%	49//59//18%
S + A	52//67//32%	52//68//33%	20//35//19%	20//36//19%

Additionally, we define a metric inspired by Maser et al.^12^ called the average improvement over baseline (AIB %):
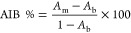
8where *A*_m_ is the exact match combination accuracy of the model and *A*_b_ is the exact match combination accuracy of choosing the top 3 most common values of a component in the respective train set.

**Figure 2:**
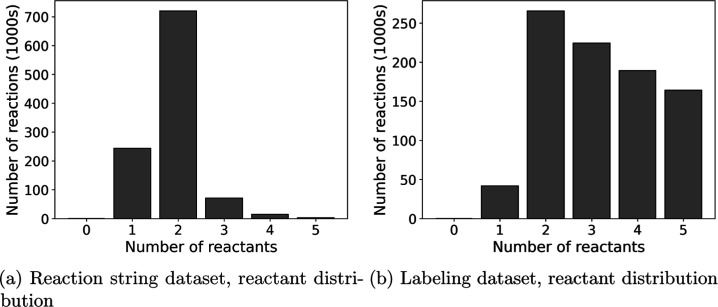
Distribution of the number of reactants between the reaction string and labeling data sets after completing other cleaning steps but not filtering out reactions with too many reactants. The data set used for these plots is therefore larger than the final condition prediction data sets. The labeling data set contains more reactants per reaction on average; this is due to agents being mislabeled as reactants.

Table 3 shows the predictive performance on the test set using four different flavors of the ORDerly-condition benchmark. All models show an improvement over the frequency informed baseline. The performance of the labeling data sets at first appears to be better than those that use our custom logic to extract reaction components from the reaction string. However, as shown in Figure 2, many of the reactions in data sets where we trust the labeling in ORD have more than three reactants, while most reactions in organic chemistry only have two reactants. Upon manual inspection, we found that many reagents and solvents were mislabeled as reactants, and therefore, the prediction problem was made significantly easier by only requiring fewer components to be predicted. In contrast, our custom cleaning pipeline that defines components using the reaction string avoided contamination of the desired prediction targets (i.e., the agents) in the inputs and, therefore, better represents the downstream application of reaction condition prediction models. This insight is confirmed in Table 4; there are fewer unique solvents and agents and a higher density of null components when using the ORD labeling instead of the reaction string, indicating that many components might be mislabeled as reactants. This discrepancy demonstrates that naive creation of data sets based on ORD can lead to inflated performance metrics.

**Table 4:** Diversity in the Data Sets^a^

	labeling			reaction string		
	(a)	(b)	(c)	(a)	(b)	(c)
reactants	207,066	0	6.95%	503,625	0	12.96%
products	253,908	0	0.0%	694,279	0	0.0%
solvents	59	598	65.84%	104	316	45.72%
agents	50	546	92.54%	275	24,547	60.93%

a Frequency filtering was applied for the solvents and agents to create a more dense one-hot encoding. (a) Number of unique molecules with a frequency above the threshold. (b) Number of unique molecules with a frequency below the threshold (Note: frequency filter only applied to solvents and agents). (c) Percentage of the component column(s), which is/are empty.

**Figure 3:**
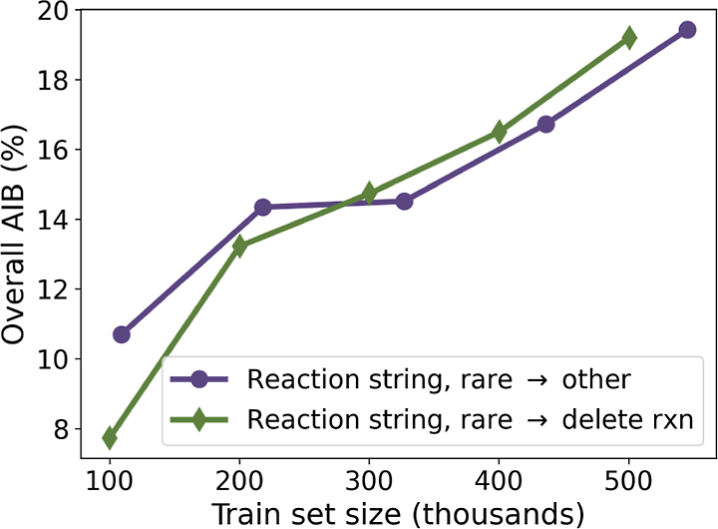
Scaling behavior of different data sets with respect to overall top-3 AIB (%) for all solvents and agents (third row from Table 3.).

For the data sets that extract the components from the reaction string, overall top-3 accuracy is only around 35% across solvents and agents. While not directly comparable, our overall accuracy is lower than what Gao et al.^11^ achieved with 50.1% top-3 accuracy across catalysts, solvents and agents. However, Gao et al. trained on approximately ten million reactions, while we train on less than 7% of that (∼691 k). As shown in Figure 3, we see consistent increases in AIB (%) with the number of data points, and this scaling performance indicates that as ORD grows, better performance could be achieved, even with potentially fewer data points than used in the paper by Gao et al.

Finally, the approach to dealing with rare values is investigated. The reaction string data sets would have more than 24,000 unique agents (see Table 4) with no frequency based filtering, which would create a sparse OHE. We initially hypothesized that the rare → “other” strategy would allow for better generalization, since the edge case reactions would be kept in a way that also keeps the OHE at a reasonable size. This behavior was indeed observed at small data set sizes (100–200 k), but as the data set size grew, the two strategies for handling rare solvents and agents performed similarly, as seen in Figure 3.

Four data sets for condition prediction were presented, varying in their handling of rare solvents and agents, and how reaction roles were assigned. The data set chosen as the ORDerly benchmark for condition prediction (ORDerly-condition) assigned reaction roles using the reaction string and used the rare → delete rxn strategy for rare spectator molecules, since this combination exhibited good accuracy, matching the usual methodology for handling rare conditions, with minimal data leakage.

### Computational Details

These models were trained on an A10G cloud GPU instance provided by lightning.ai for 100 epochs to minimize cross-entropy loss for each reaction component. The best model based on validation loss was chosen for evaluation.

### Component Labeling

There are two ways of assigning reaction roles to molecules found in ORD files, either relying on the labeling or identifying reaction roles by considering the atom mapping of a reaction SMILES string. We found that relying on the labeling in ORD mislabels many spectator molecules as reactants, which explains the difference in reactant count distribution seen in Figure 2. Identifying the role of molecules in a reaction provides crucial context to machine learning models, adding domain knowledge to the data, thereby improving performance. Atom mapping the reactions with the newest algorithm may allow for greater accuracy in identifying reaction roles,^52^ however, an atom mapping algorithm was not integrated into ORDerly to keep ORDerly lightweight. With the existing atom mapping in ORD, molecules contributing atoms to the product could readily be bundled together and labeled as reactants. However, subdividing spectator molecules into different categories (e.g., agents, reagents, solvents, catalysts, precatalysts, ligands, acids/bases) is a difficult task. The difficulty is compounded by the fact that the same molecule can play different roles, depending on the context. The role that a molecule plays in a reaction may more easily be identified when only considering one reaction class at a time,^12^ since this allows the mechanistic details of the reaction class^53−55^ to be considered. Handling large and diverse data sets inevitably requires generalizations that may result in contradictions upon a more fine-grained inspection. In this work, solvents were separated from the other spectator molecules because these can somewhat reliably be identified. Catalysts were not separated into their own category since identifying catalysts is more subtle (especially with organocatalysis), and few reactions in the reaction string data sets contained transition metals.

### Order Invariance

Although the order of addition may play a role in wet lab chemistry, reaction prediction tasks are often cast as order invariant, where the goal is to predict a set of molecules. However, both of the architectures used for in silico validation of the ORDerly data sets are not agnostic to the ordering of the targets, since the neural networks used predict one molecule at a time in the OHE, and the transformers used predict one token at a time. Incorporating order invariance (and canonicalization) of molecules into the loss calculation during training may allow for better generalizability of predictive models and is an exciting area for further study. It is worth noting that the evaluation metrics used throughout are order invariant.

## Conclusions

In this work, we presented ORDerly, an open-source framework for preparing chemical reaction data stored in the Open Reaction Database (ORD) for machine learning applications. ORDerly was used to generate benchmark data sets for forward prediction (ORDerly-forward), retrosynthesis (ORDerly-retro), and condition prediction (ORDerly-condition) based on US patent data. Transformer models were trained on the forward prediction and retrosynthesis data sets, and they were found to only generate invalid SMILES strings very infrequently, while also achieving similar test accuracy to that found in the literature on a held-out set of US patents. ORDerly was also used to generate test sets from all nonpatent data from ORD, which could serve as a better indication of model generalization when potential mislabeling does not interfere with the prediction task. The condition prediction task was used to investigate different strategies for assigning reaction roles and frequency filtering of spectator molecules. When building data sets for condition prediction using the labeling in ORD, we found contamination of the inputs (reactants) with the outputs (agents), resulting in a problem that was unrealistically easy. We therefore chose to use chemically informed logic to better assign reaction roles for the ORDerly-condition benchmark.

All benchmarks and data sets experimented with in this work, as well as the code used to generate them, are freely available online (see Data Availability Statement), and we hope the benchmarks will make reaction prediction tasks more accessible to ML practitioners with limited domain knowledge. ORDerly presents a fully open-source pipeline to go from raw ORD data to a fully trained condition prediction model, allowing for an avenue to leverage the growing contributions to open-source chemistry.

## Data Availability

The ORDerly python package is released under the MIT license, and is available at https://github.com/sustainable-processes/orderly. All data sets are released under the CC BY 4.0 license; the ORDerly benchmark data sets are available for download at https://figshare.com/articles/dataset/ORDerly-chemical_reactions_condition_benchmarks/23298467, and all other data sets mentioned are available for download at https://figshare.com/articles/dataset/ORDerly_datasets/23502372.

## Supporting Information Available

The Supporting Information contains further details on the in silico experiments, package documentation, data set statistics and datasheet, and example reactions from ORDerly-condition (PDF)

## Footnotes

a We discuss the difference between these data sets and our data sets in the Supporting Information.
