# Supplementary material for: ORDerly: Data Sets and Benchmarks for Chemical Reaction Data
Source: J Chem Inf Model. 2024 Apr 22;64(9):3790–8. doi: 10.1021/acs.jcim.4c00292 (PMC11094788; doi:10.1021/acs.jcim.4c00292)
Supplement: Supplementary file 1 — ci4c00292_si_001.pdf [file ci4c00292_si_001.pdf]

Supporting Information for

# ORDerly: Datasets and Benchmarks for Chemical Reaction Data

Daniel S. Wigh, Joe Arrowsmith, Alexander Pomberger, Kobi C. Felton, and Alexei A. Lapkin*

*Department of Chemical Engineering and Biotechnology, University of Cambridge, Cambridge CB3 0AS, UK*

E-mail: aal35@cam.ac.uk

## Problem Formulation

As noted by Meng *et al.*,[1] reaction related tasks operate on molecules. There are numerous machine readable molecular representations,[2] including molecular graphs and strings, and in this work molecules are represented as SMILES strings. Each character $m_i$ in a SMILES string represents an atom or a molecular feature (bond, branch, ring closure): $\mathcal{M} := m_1, m_2, m_3, \ldots, m_L$, where $L$ is the total number of characters in the string. Molecules can take on one of three roles in a reaction: reactant, product, or agent. A reaction $\mathcal{R}$ transforms $N$ reactant molecules (sometimes called educts) $\{\mathcal{M}_i^{\mathcal{E}}\}_{i=1}^{N}$ by breaking and forming bonds to form $M$ new product molecules $\{\mathcal{M}_i^{\mathcal{P}}\}_{i=1}^{M}$ using $K$ agent molecules $\{\mathcal{M}_i^{\mathcal{A}}\}_{i=1}^{K}$. Agents are helper molecules that enable the reaction to proceed (e.g., solvents, catalysts).

$$\mathcal{R} : \{\mathcal{M}_i^{\mathcal{E}}\}_{i=1}^{N}, \{\mathcal{M}_i^{A}\}_{i=1}^{K} \rightarrow \{\mathcal{M}_i^{P}\}_{i=1}^{M}, \{\mathcal{M}_i^{A}\}_{i=1}^{K} \quad (1)$$

Given this view of reactions, we define four different reaction related tasks in this work.

**Forward prediction** is the task of predicting the product of a reaction $\mathcal{M}^{\mathcal{P}}$ given its reactants $\{\mathcal{M}_i^{\mathcal{E}}\}_{i=1}^{N}$ and, potentially, agents $\{\mathcal{M}_i^{\mathcal{A}}\}_{i=1}^{K}$. Probabilistically, the task is to predict the distribution $p(\mathcal{M}^{\mathcal{P}}|\{M_i^{\mathcal{E}}\}_{i=1}^{N})$. While experimental evaluation in a wet lab requires expert chemists and is a time intense task, reaction outcome prediction can help as a tool to evaluate the quality of a predicted retrosynthetic route (i.e., the probability that the reaction predicted by the single-step retrosynthesis model leads to the desired product).[3]

**Retrosynthesis** is the task of designing a sequence of $Z$ reactions $\mathcal{R}_1, \mathcal{R}_2, \mathcal{R}_3, \ldots, \mathcal{R}_Z$ that transform a set of readily available reactant molecules $\{\mathcal{M}_i^{\mathcal{E}_1}\}_{i=1}^{N}$ to a desired product(s) $\{\mathcal{M}_i^{\mathcal{P}_Z}\}_{i=1}^{M_Z}$. Retrosynthesis is done in the reverse direction by starting with the desired product(s) $\{\mathcal{M}_i^{\mathcal{P}_Z}\}_{i=1}^{M_Z}$ and predicting reactants $\{\mathcal{M}_i^{\mathcal{E}_Z}\}_{i=1}^{N_Z}$ that would react to form the desired product(s). The predicted reactants $\{\mathcal{M}_i^{\mathcal{E}_Z}\}_{i=1}^{N_Z}$ then become the products of the next reaction to be predicted $\{\mathcal{M}_i^{\mathcal{P}_{Z-1}}\}_{i=1}^{M_{Z-1}}$. This process is repeated until a readily available set of starting reactant molecules are predicted $\{\mathcal{M}_i^{\mathcal{E}_1}\}_{i=1}^{N}$. Therefore, the key machine learning task, often called single-step retrosynthesis, is predicting the distribution $p(\{\mathcal{M}_i^{\mathcal{E}_j}\}_{i=1}^{N_j})|\mathcal{M}^{P_j})$ or the set of reactants that could lead to a given product(s) $\{\mathcal{M}_i^{\mathcal{P}_j}\}_{i=1}^{M_j}$. Single-step retrosynthesis can be seen as the inverse of forward prediction.

**Condition prediction** is the task of predicting the distribution $p(\{\mathcal{M}_i^{\mathcal{A}}\}_{i=1}^{K}|\{M_i^{\mathcal{E}}\}_{i=1}^{N}, \mathcal{M}^{\mathcal{P}})$ (i.e., the agents for a reaction given reactants and product). In addition to agents, some models can predict continuous variables such as reaction temperature and concentrations of reactants and agents.[4]

**Yield prediction** is the task of predicting the percentage yield ($y$) of the product relative to the limiting reactant achieved upon running a reaction to completion $p(y|\{M_i^{\mathcal{E}}\}_{i=1}^{N}, \mathcal{M}^{\mathcal{P}}, \{\mathcal{M}_i^{\mathcal{A}}\}_{i=1}^{K})$. Yield prediction models trained on high-throughput experimentation data generally perform much better than models trained on literature and/or patent data.[5,6]

# Additional Dataset

We used ORDerly to generate an additional dataset, `ORDerly-condition-with-rare`. This dataset can be found on the same FigShare page as the other datasets. `ORDerly-condition` is built from USPTO, and has had rare solvents and agents (appearing less frequently than 100 times) removed. `ORDerly-condition-with-rare` is built and cleaned in the same way as `ORDerly-condition`, except it keeps the rare solvents and agents.

ORDerly was not used to create a benchmark for yield prediction from USPTO due to known issues with patent yield data. The yield data in USPTO is noisy, and the distribution of yields differs depending on the mass scale. Good performance in yield prediction is generally only possible with data from high throughput experiments.[5]

Table S1: Number of reactions left in each dataset after cleaning.

| Dataset name | `ORDerly-condition` | `ORDerly-condition-with-rare` |
|---|---|---|
| Full dataset | 1,771,032 | 1,771,032 |
| Too many reactants | 1,627,929 | 1,627,929 |
| Too many products | 1,589,977 | 1,589,977 |
| Too many solvents | 1,385,579 | 1,385,579 |
| Too many agents | 1,279,207 | 1,279,207 |
| No reactants/products | 1,261,701 | 1,261,701 |
| Inconsistent yield | NA | NA |
| Dropping duplicates | 753,338 | 753,338 |
| Frequency filtering | 691,142 | NA |

# Dataset Extraction and Cleaning

In the paper, we describe the "labeling" and "reaction string" datasets; in the code this is denoted by `trust_labeling=True`, and `trust_labeling=False`, respectively. We also presented two different strategies for dealing with rare molecules, either "rare→"other" and "rare→delete rxn", these are denoted in the code as `map_rare_molecules_to_other=True`, and `map_rare_molecules_to_other=False`, respectively. There are a number of other

tuneable parameters in the scripts, and below we explain how default values were chosen for each of these.

## Extraction Script

There are three fields in the Open Reaction Database schema to extract molecules from: the input, the outcome, and the reaction string. Molecules in the reaction string are represented as SMILES, while molecules in the input and outcome field can be represented with a number of different representations, including SMILES, InChI, and plain text English names. When extracting molecules from the input or the outcome fields, the preferred representation was SMILES. However, how should the situation where only an English name exists be dealt with? It is tempting to check whether the representation is interpretable by RDKit (potentially implying that the molecular representation was mislabeled as a name rather than SMILES), however, this can lead to unexpected behaviour. As an example, the string, "1400C", was encountered as the name for a molecule, should this be interpreted as a graphene structure, a typo for carbon-14, a typo for 1400°C, or simply carbon? Another situation which was encountered was BOC; this *is* a resolvable SMILES string, representing boron oxygen and carbon bonded together, however, in context, it was actually referring to a BOC group (tert-butyloxycarbonyl protecting group). Another example of unintended behaviour is the case of II, which could mean diiodine, but also mark the second step/item when counting. Therefore, when users decide not to trust the labeling of molecules, molecules only represented with a plain text name were ignored, to avoid ambiguity.

The extraction script generates relevant data from each ORD file, and allows for the following customization:

Note that we only mention the arguments that materially affect the science/logic of how cleaning is done.

- `trust_labeling`: If True, maintain the labeling of the data in ORD. If False: chemical logic (described extensively in the paper) is applied to the reaction string to determine

the reaction role of molecules.

- solvents_path: If a user does not trust the labeling, all agent molecules are cross-checked against a set of industrially relevant solvents (compiled by us from three different sources), and any matches are re-labeled as solvents. See the Building a Set of Solvents section for how this set of solvents was constructed.

- name_contains_substring: Only extract filenames from ORD that includes this string. If left empty will not search for anything, and if set to None it will extract data from all ORD files in the designated folder. For example, setting name_contains_substring="uspto" will grab all files that have "uspto" in the file name (i.e. the USPTO data).

- inverse_substring: The inverse of name_contains_substring, e.g. setting inverse_substring="uspto" will grab everything *except* the USPTO data.

**Building a Set of Solvents**

The solvents set can be found in orderly/data/solvents.csv in the ORDerly GitHub repository. The set was created from the union of solvents from the following three sources:

- Machine learning and molecular descriptors enable rational solvent selection in asymmetric catalysis (458 solvents)[7]

- ACS Green Chemistry: Solvent Selection Tool (272 solvents)[8]

- Summit GitHub Repository (115 solvents)[9]

After the data from these three sources were concatenated into a new CSV files, the solvents were filtered by: making all solvent names lower case, stripping spaces, and then removing duplicate names. (Before removing duplicates: 458+272+115=845 solvents. After removing duplicates: 615 solvents.) Then Pura[10] was run to resolve the solvent name where no SMILES string was available. Each solvent (with no SMILES string) was represented with

up to four different names: three English solvent names (synonym names) and one CAS number. Pura was used with `services=[PubChem(autocomplete=True), Opsin(), CIR()]` and `agreement=2` on each English name, and `services=[CAS()]` with `agreement=1` on the CAS number. This yielded up to four different SMILES strings for each solvent. SMILES strings with full agreement for a solvent were trusted, and any rows with disagreement between the SMILES strings ($\approx 40$ solvents) were resolved by hand. The final solvents set is a CSV file with seven columns: up to three different English solvent names (synonyms), a CAS number, a chemical formula, SMILES, and finally the source.

An obvious drawback of identifying solvents by crosschecking against a curated set is that the set naturally will be incomplete; there are unfathomably many different organic molecules, and it is unclear how many of these could act as solvents. However, not distinguishing between solvents and agents may make the learning task more difficult for machine learning models, and using the labeling that already exists in ORD was routinely found to be inaccurate. In practice, the vast majority of solvents used in industry and academia are inspired by what has previously been proven successful, and thus the solvents set curated for this work is likely going to capture a majority of solvents that are routinely used. Another difficulty is that the role of solvent molecules may depend on context (e.g. polar protic solvents may contribute protons to a product, in which case the role of the molecule becomes murky (i.e. is it a reactant since it contributed atoms to the product, is it a solvent since it dissolved the (other) reactants, or is it a reagent since it acts like an acid?).

## Cleaning Script

- `remove_reactions_with_no_reactants [bool]`: Self-explanatory
- `remove_reactions_with_no_products [bool]`: Self-explanatory
- `consistent_yield [bool]`: If True, removes reactions that have yields that do not make sense, e.g. if any individual yields, or the sum of yields, is outside of [0%; 100%]

(reactions with no yields are kept).

- num_reactant, num_product, num_solv, num_agent, num_cat, num_reag [int]: The maximum number of components allowed of the specified type in a reaction. E.g. if num_solv=2 any reactions with 3 or more solvents will be dropped from the DataFrame. See the ORDerly Benchmark Statistics section for how the default values were chosen.

- min_frequency_of_occurrence [int]: The minimum frequency of solvents and agents for the reaction to be kept. The frequency of molecules across all columns of the same type (e.g. solvents) are counted, and any reactions containing molecules below the frequency cutoff are dealt with in accordance with map_rare_molecules_to_other. See the ORDerly Benchmark Statistics section for how the default values were chosen.

- map_rare_molecules_to_other [bool]: If False, any reactions containing molecules that fall below the threshold will be deleted. If True, the rare molecules will be mapped to a string "other", allowing us to keep the reactions in the dataset. This behaviour can be shut off simply by setting min_frequency_of_occurrence=0.

- set_unresolved_names_to_none_if_mapped_rxn_str_exists_else_del_rxn, remove_rxn_with_unresolved_names, set_unresolved_names_to_none [bool]: These three bools control the handling of unresolvable names (i.e. names that are unresolvable by RDKit, and do not exist in our manually curated name resolution dictionary, and at most one of them can be True (if all are set to False, unresolvable names are kept in the dataset.) While the second and third bool are self-explanatory, this is the logic applied if the first bool is True: if a reaction contains a mapped reaction, the reaction is seen as quite trustworthy, and therefore the unresolvable names can safely be set to None, while the remaining data associated with that reaction is maintained; if a reaction does not have an associated mapped reaction, the presence of an unresolveable name is a red flag casting doubt on the veracity of that reaction, and thus the whole reaction (a row in the DataFrame) is removed).

## Further Justification for Cleaning Thresholds

### Condition Prediction Benchmark

- **Reactant filtering:** Reactions with more than two reactants were filtered out, since they are likely to be multi-step reactions or complex one-pot reactions (tri-molecular mechanisms are exceedingly rare in chemistry).
- **Product filtering:** Reactions with multiple products were also filtered out since nearly all reactions in USPTO only report one product (see Figure AS1); predicting reaction side products and impurities remains an active area of research,[11] and thus fell beyond the scope of ORDerly.
- **Solvent and agent filtering:** Thresholds for the number of spectator molecules was set at two solvents and three agents to have the same number of categorical variables as in the model of Gao *et al.*[4]
- **Not predicting temperature:** Only 293k out of 626k reactions in the `ORDerly-condition` training set contain a temperature, of which over half report 25C. Filtering away reactions without a temperature record would leave a much smaller dataset, and we do not believe that it is reasonable to assume that reactions without a reported temperature were performed at room temperature.

### Forward Prediction and Single-step Retrosynthesis benchmarks

The `ORDerly-retro` dataset is compared to other standard forward prediction and retrosynthesis datasets in Table S2. USPTO-50K was created by Schneider *et al.* for testing reaction role assignment.[12] They used NameRxn to assign reaction classes to all the reactions in the dataset. Liu *et al.*[13] then used the USPTO-50K for benchmarking their retrosynthesis model, however, they did not use the reaction classes to create a split, and instead opted for a random split. Coley *et al.*[14] is often cited for their train/test split of USPTO-50K.

USPTO-MIT is a larger set that was introduced by Jin *et al.*[15]

Table S2: A comparison between different datasets for retrosynthesis and forward prediction. Note that for USPTO-full the original paper states there are 1.9M reactions, but we found less than 1M as of August 2023. The unprocessed USPTO dataset in ORD consists of 1.77M reactions.

| Dataset | Size | Split | Reference |
|---|---|---|---|
| USPTO-50K | 50 016 | Random | [12] |
| USPTO-MIT | 479 035 | Random | [15] |
| USPTO-full | 997 415 | Random | [16] |
| `ORDerly-retro` | 941 566 | Random | This work |
| `ORDerly-forward` | 919 231 | Random | This work |

- **Forward prediction:** A small number of reactions in USPTO reported two products, and for the forward prediction dataset we allowed up to two products and three reactants, solvents, and agents.
- **Retrosynthesis prediction:** In retrosynthesis prediction the goal is to predict reactants that can be used to form a desired product. To ensure that the difficulty of the task was reasonable, we limit reactions to having one product and two reactants, such that the models only have to learn how to break one molecule into two, and not consider e.g. multi-producut or multi-step reactions. Only product and reactant molecules were used in the retrosynthesis dataset, so there were no restrictions in the number of solvents and agents.

# Further Experimental Details

## Condition Prediction with Neural Networks

The code from Gao *et al.*[4] was used for training condition prediction models. The hyperparameters in Table S3 were used, which reflect those used in the original paper. The models were trained on an A10G cloud GPU instance provided by lightning.ai for 100 epochs to

minimize cross entropy loss for each reaction component, taking 30 min or less for a full training run. The best model by validation loss was chosen for evaluation.

Table S3: Hyperparameters used for training condition prediction models

| | |
|---|---|
| batch size | 512 |
| learning rate | 0.01 |
| hidden size 1 | 1024 |
| hidden size 2 | 100 |
| dropout | 0.2 |
| fingerprint size | 2048 |

### Forward Prediction and Retrosynthesis Prediction with Transformers

Most of the hyperparameters used in the Molecular Transformer architecture (see Table S4) were the defaults suggested by Schwaller *et al.*[17] (GitHub: `https://github.com/pschwllr/MolecularTransformer`). The transformer models were trained on Tesla T4 GPUs provided by lightning.ai for 500 000 steps (roughly 1000 epochs, taking around 70 hours each).

## ORDerly Benchmark Statistics

### Number of Components

Figures S1 and S2 shows the distribution in the number of components of the unfiltered datasets, allowing us to compare the reaction string datasets to the labeling datasets. The distributions look quite similar for products and solvents. However, the distributions are different for reactants and agents/catalysts, which can be explained by reagents routinely being labeled as reactants in ORD.

Table S4: Hyperparameters for Molecular Transformer.

| | | |
|---|---|---|
| Training | | |
| | seed | 42 |
| | param_init | 0 |
| | param_init_glorot | |
| | max_generator_batches | 32 |
| | batch_size | 4096 |
| | batch_type | tokens |
| | normalization | tokens |
| | max_grad_norm | 0 |
| | accum_count | 4 |
| | optim | adam |
| | adam_beta1 | 0.9 |
| | adam_beta2 | 0.998 |
| | decay_method | noam |
| | warmup_steps | 8000 |
| | learning_rate | 2 |
| | label_smoothing | 0.0 |
| | layers | 4 |
| | rnn_size | 256 |
| | word_vec_size | 256 |
| | encoder_type | transformer |
| | decoder_type | transformer |
| | dropout | 0.1 |
| | position_encoding | |
| | share_embeddings | |
| | global_attention | general |
| | global_attention_function | softmax |
| | self_attn_type | scaled-dot |
| | heads | 8 |
| | transformer_ff | 2048 |
| Inference | | |
| | batch_size | 512 |
| | replace_unk | |
| | max_length | 200 |
| | beam_size | 5 |

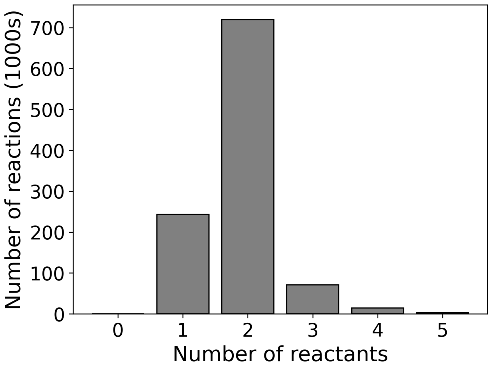


(a) Reaction string dataset, reactant filtering

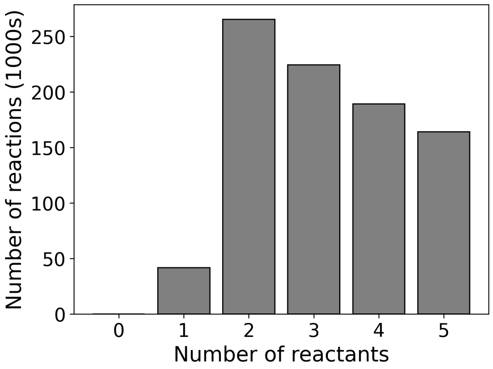


(b) Labeling dataset, reactant filtering

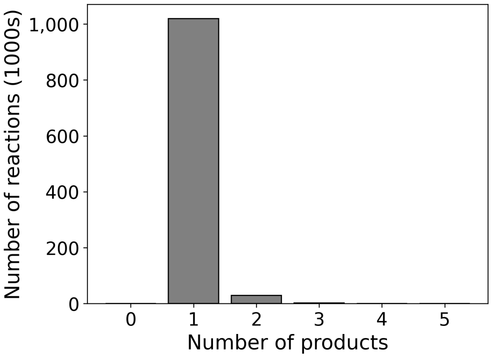


(c) Reaction string dataset, product filtering

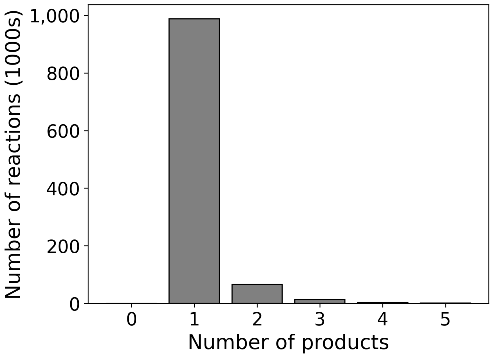


(d) Labeling dataset, product filtering

Figure S1: Distribution of the number of components between the reaction string and labeling datasets for reactants and products when not filtering out reactions with too many of a certain component (e.g. too many reactants). The dataset used for these plots is therefore larger than the final datasets.

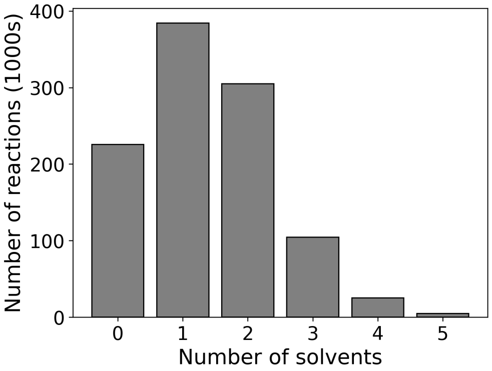


(a) Reaction string dataset, solvent filtering

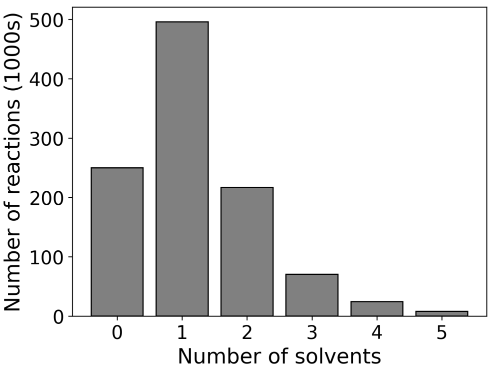


(b) Labeling dataset, solvent filtering

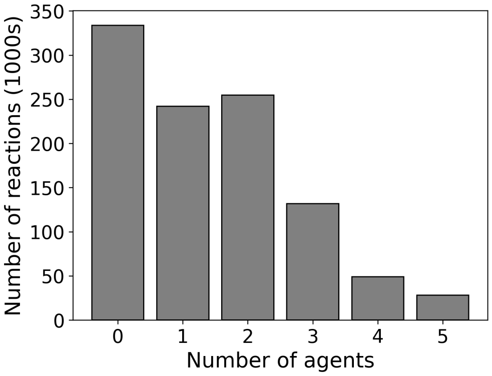


(c) Reaction string dataset, agent filtering

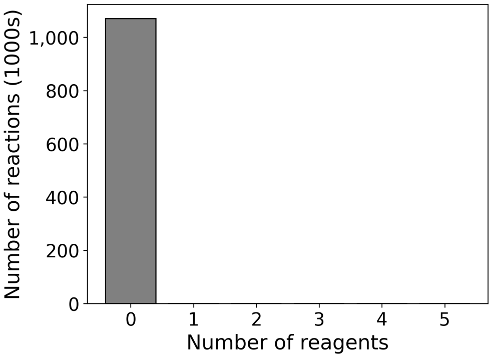


(d) Labeling dataset, reagent filtering

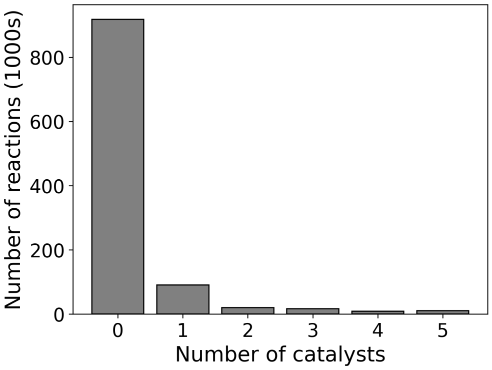


(e) Labeling dataset, catalyst filtering

Figure S2: Distribution of the number of components between the reaction string and labeling datasets for spectator molecules (agents, solvents, catalysts, reagents) when not filtering out reactions with too many of a certain component (e.g. too many solvents). The dataset used for these plots is therefore larger than the final datasets. There are no reagents in the labeling dataset, so after filtering, excess catalysts were re-labeled as reagents.

## Minimum Frequency of Occurrence

Figure S3 shows how many reactions would be left in the reaction string and labeling datasets as a function of the minimum frequency of occurrence. The minimum frequency of occurrence is the threshold applied to the spectator molecules (solvents, agents, reagents, agents, catalysts) to be considered rare, and any reactions containing a rare molecule will be deleted if the "rare→delete rxn" strategy is used.

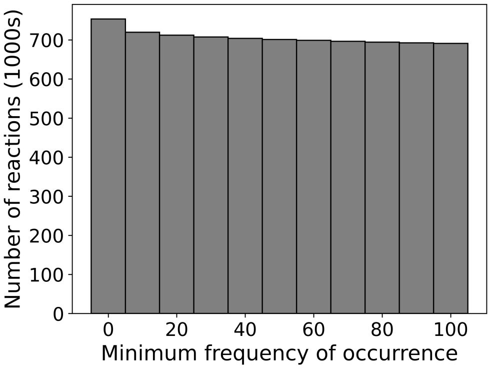


(a) Reaction string dataset (x: 10-100)

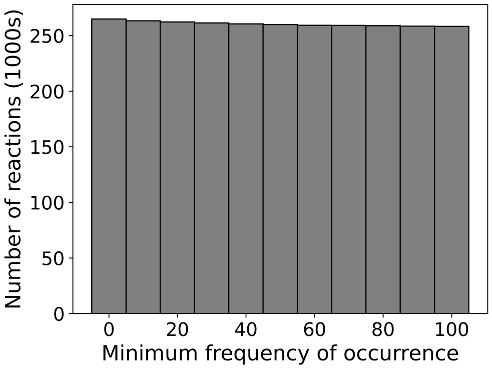


(b) Labeling dataset (x: 10-100)

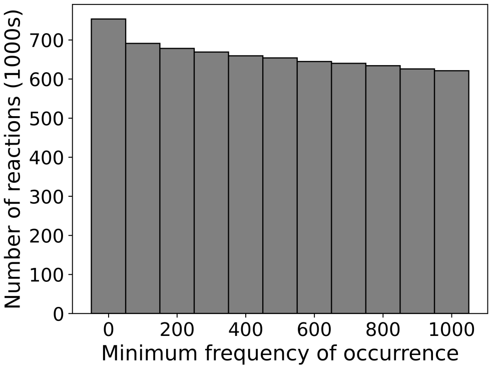


(c) Reaction string dataset (x: 100-1000)

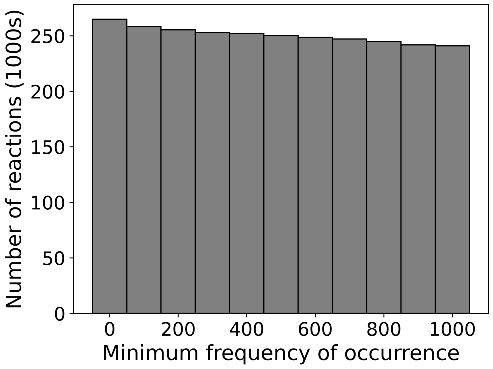


(d) Labeling dataset (x: 100-1000)

Figure S3: Impact on dataset size by changing the minimum frequency of occurrence.

## Molecule Popularity

Figures S4 and S5 shows the distribution of occurrence of the top 100 most popular molecules across the different categories of molecules for the labeling and rxn string datasets. Across categories, the reaction string dataset is more diverse and not as heavily dominated by the

most popular component. It is also interesting that the most popular molecules between the datasets are not the same, despite being based on the same raw data.

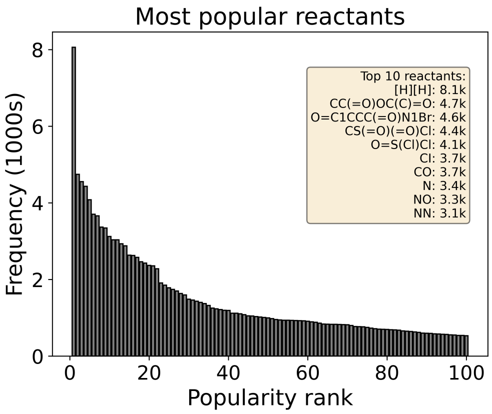


(a) Reaction string dataset, reactants

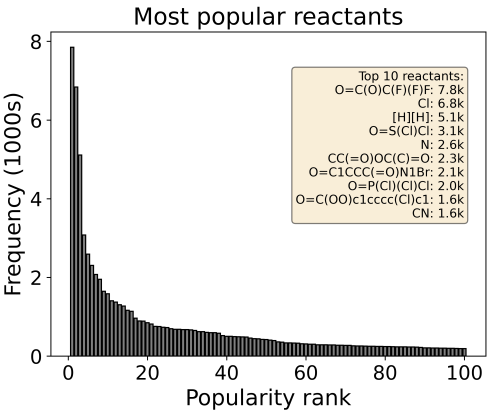


(b) Labeling dataset, reactants

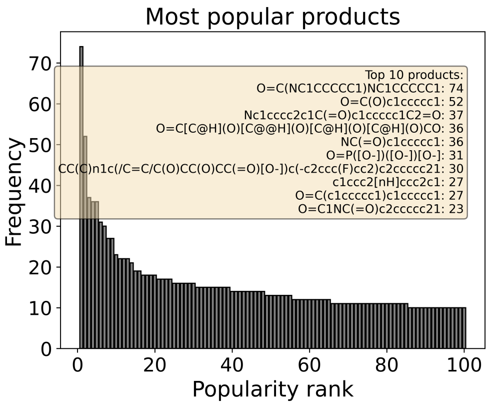


(c) Reaction string dataset, products

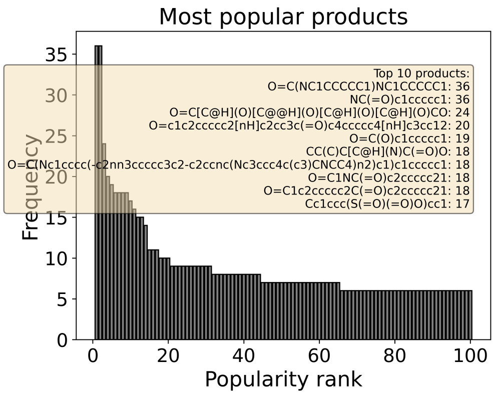


(d) Labeling dataset, products

Figure S4: Frequency of occurrence of the most popular molecules for reactants and products. NULL has been removed.

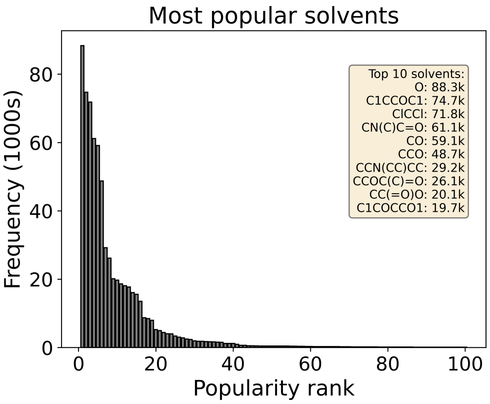


(a) Reaction string dataset, solvents

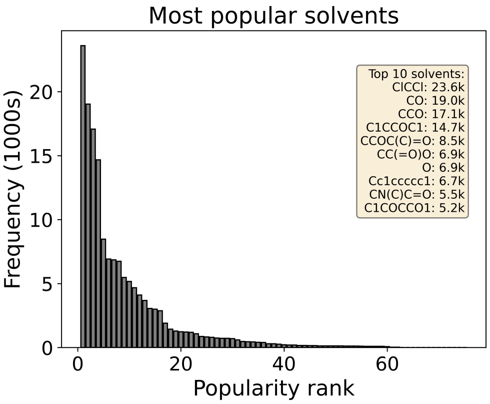


(b) Labeling dataset, solvents

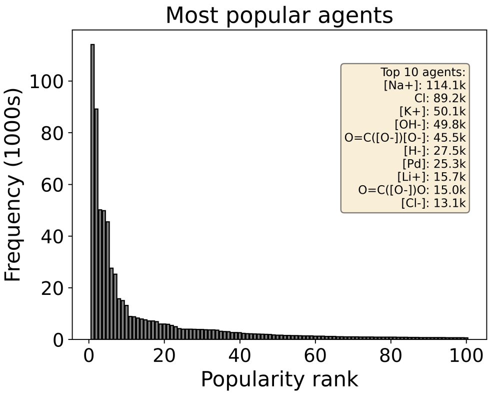


(c) Reaction string dataset, agents

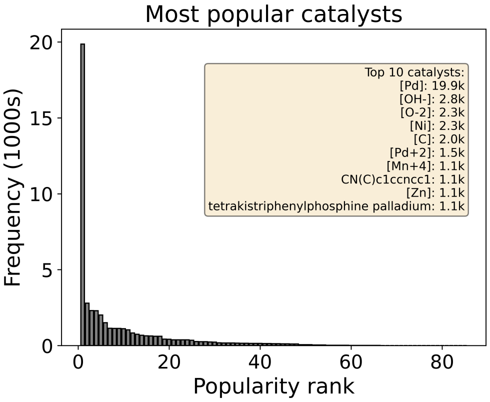


(d) Labeling dataset, catalysts

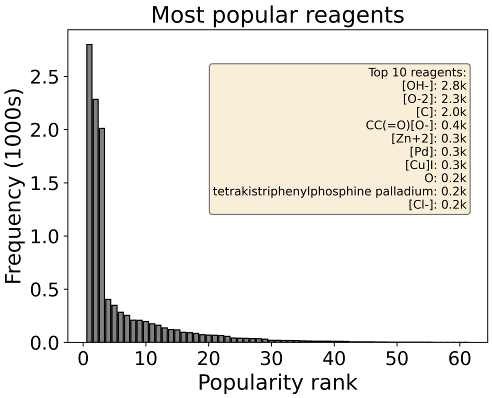


(e) Labeling dataset, reagents

Figure S5: Frequency of occurrence of the most popular molecules for spectator molecules (agents, reagents, solvents, catalysts). NULL has been removed.

# Example Reaction Instances and Predictions

In this section, we give examples of reactions that are in both the trust labeling and reaction string datasets to demonstrate the differences between the datasets. Note that the leakage between model inputs (reactants) and model outputs (solvents, reagents) in the labeling dataset leads to inflated accuracy of the model trained on the labeling dataset. This leakage comes from the reagents often being mislabeled as reactants, as apparent from the difference in reactant distribution seen in Figure S1, and also from the diversity of the labeling dataset being lower, as apparent from Figure S5. Furthermore, many reactions contain no solvents or agents, leading to the model trained on the labeling dataset to over-predict None.

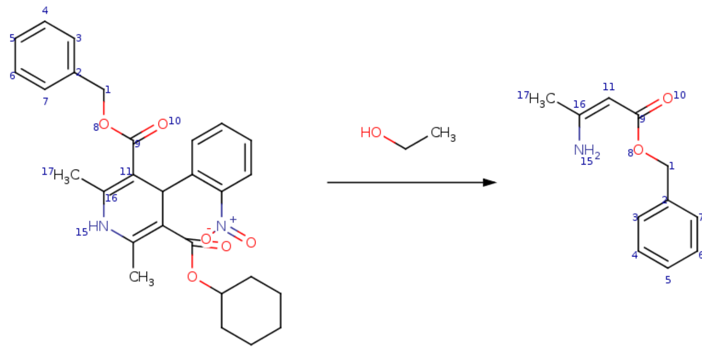


**Reaction string:**
[CH2:1]([O:8][C:9]([C:11]1C(C2C=CC=CC=2[N+]([O-])=O)C(C(OC2CCCCC2)=O)=C(C)[NH:15][C:16]=1[CH3:17])=[O:10])[C:2]1[CH:7]=[CH:6][CH:5]=[CH:4][CH:3]=1>C(O)C>[CH2:1]([O:8][C:9](=[O:10])/[CH:11]=[C:16](\[NH2:15])/[CH3:17])[C:2]1[CH:7]=[CH:6][CH:5]=[CH:4][CH:3]=1

| | **Reaction string dataset (This work)** | **Trust labelling dataset** |
|---|---|---|
| Reactants | 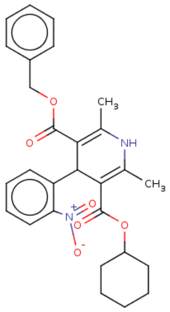 | 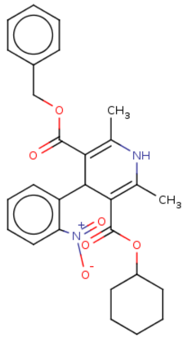 |
| Products | 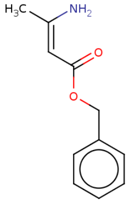 | 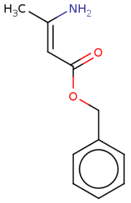 |
| Ground truth solvents | 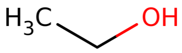 | 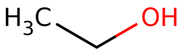 |
| Ground truth agents | None | None |
| Predicted solvents | 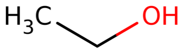 ✓ | 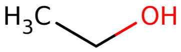 ✓ |
| Predicted agents | None ✓ | None ✓ |

Comment: Correct classification of the reactants. Ethanol can be seen either as reagent or solvent. Agents were marked as None for both datasets.

✓ Correct prediction ✗ Incorrect prediction ✓ Partially correct prediction

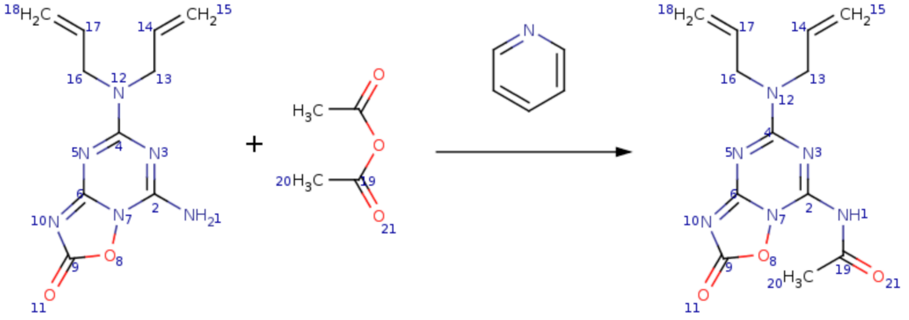

**Reaction string:**

[NH2:1][C:2]1[N:7]2[O:8][C:9](=[O:11])[N:10]=[C:6]2[N:5]=[C:4]([N:12]([CH2:16][CH:17]=[CH2:18])[CH2:13][CH:14]=[CH2:15])[N:3]=1.[C:19](OC(=O)C)(=[O:21])[CH3:20]>N1C=CC=CC=1>[CH2:13]([N:12]([CH2:16][CH:17]=[CH2:18])[C:4]1[N:3]=[C:2]([NH:1][C:19](=[O:21])[CH3:20])[N:7]2[O:8][C:9](=[O:11])[N:10]=[C:6]2[N:5]=1)[CH:14]=[CH2:15]

| | **Reaction string dataset (This work)** | **Trust labelling dataset** |
|---|---|---|
| Reactants | 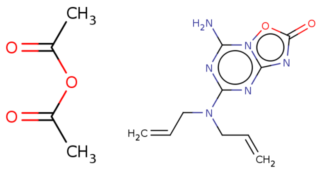 | 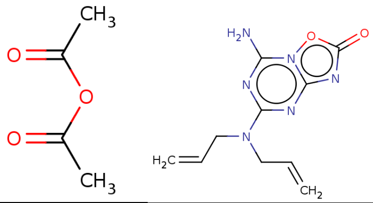 |
| Products | 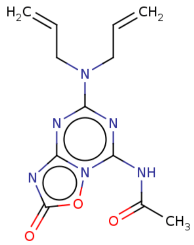 | 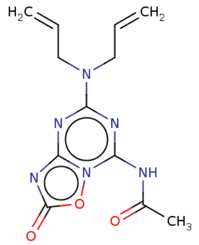 |
| Ground truth solvents | 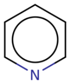 | 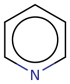 |
| Ground truth agents | None | None |
| Predicted solvents | 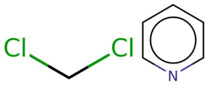 ✓ (partially correct) | 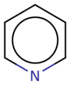 ✓ |
| Predicted agents | None ✓ | None ✓ |

Comment: Correct classification of the reactants. Partially correct for the solvent prediction. Agents were marked as None for both datasets.

✓ Correct prediction ✗ Incorrect prediction ✓ Partially correct prediction

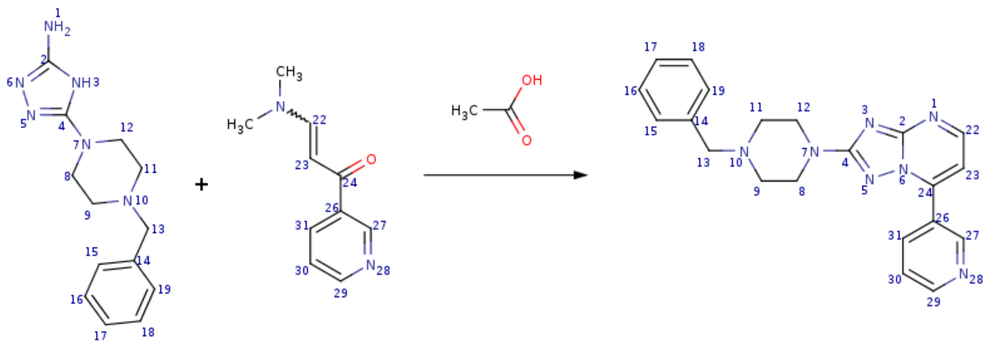

**Reaction string:**

[NH2:1][C:2]1[NH:3][C:4]([N:7]2[CH2:12][CH2:11][N:10]([CH2:13][C:14]3[CH:19]=[CH:18][CH:17]=[CH:16][CH:15]=3)[CH2:9][CH2:8]2)=[N:5][N:6]=1.CN(C)[CH:22]=[CH:23][C:24]([C:26]1[CH:27]=[N:28][CH:29]=[CH:30][CH:31]=1)=O>C(O)(=O)C>[CH2:13]([N:10]1[CH2:11][CH2:12][N:7]([C:4]2[N:3]=[C:2]3[N:1]=[CH:22][CH:23]=[C:24]([C:26]4[CH:27]=[N:28][CH:29]=[CH:30][CH:31]=4)[N:6]3[N:5]=2)[CH2:8][CH2:9]1)[C:14]1[CH:15]=[CH:16][CH:17]=[CH:18][CH:19]=1

| | Reaction string dataset (This work) | Trust labelling dataset |
|---|---|---|
| Reactants | 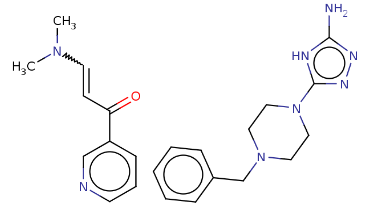 | 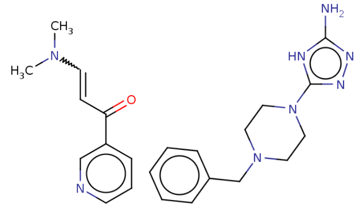 |
| Products | 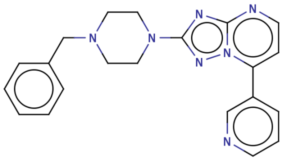 | 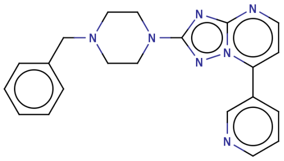 |
| Ground truth solvents | 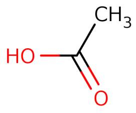 | 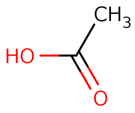 |
| Ground truth agents | None | None |
| Predicted solvents | 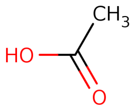 ✓ | 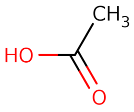 ✓ |
| Predicted agents | None ✓ | None ✓ |

Comment: Correct classification of the reactants. Correct classification of the reactant. Agents were marked as None for both datasets.

✓ Correct prediction ✗ Incorrect prediction ✓ Partially correct prediction

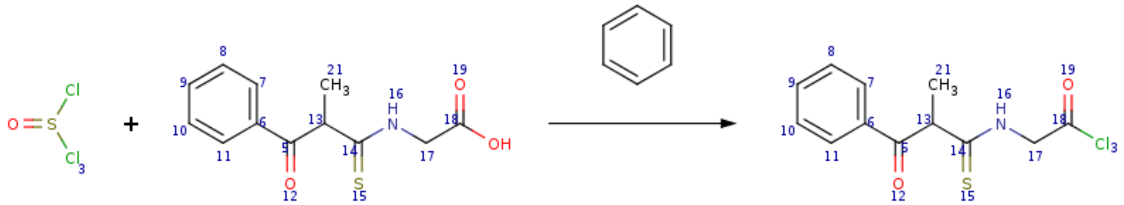

**Reaction string:**
S(Cl)([Cl:3])=O.[C:5]([CH:13]([CH3:21])[C:14]([NH:16][CH2:17][C:18](O)=[O:19])=[S:15])(=[O:12])[C:6]1[CH:11]=[CH:10][CH:9]=[CH:8][CH:7]=1>C1C=CC=CC=1>[C:5]([CH:13]([CH3:21])[C:14]([NH:16][CH2:17][C:18]([Cl:3])=[O:19])=[S:15])(=[O:12])[C:6]1[CH:11]=[CH:10][CH:9]=[CH:8][CH:7]=1

| | **Reaction string dataset (This work)** | **Trust labelling dataset** |
|---|---|---|
| Reactants | 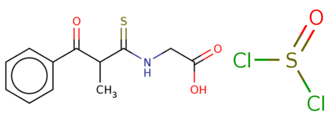 | 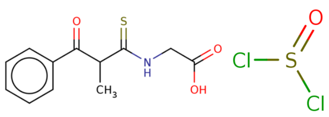 |
| Products | 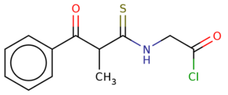 | 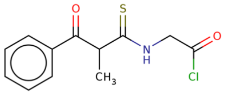 |
| Ground truth solvents | 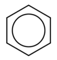 | 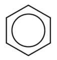 |
| Ground truth agents | None | None |
| Predicted solvents | 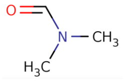 ✗ | None ✗ |
| Predicted agents | None ✓ | None ✓ |

Comment: Correct classification of the reactants. None of the two models was able to successfully deliver a correct prediction for the solvent. Agents were marked as None for both datasets.

✓ Correct prediction ✗ Incorrect prediction ✓ Partially correct prediction

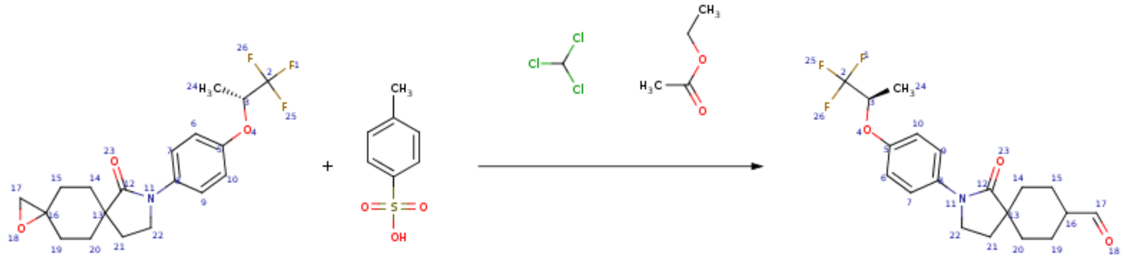

**Reaction string:**

[F:1][C:2]([F:26])([F:25])[C@@H:3]([CH3:24])[O:4][C:5]1[CH:10]=[CH:9][C:8]([N:11]2[CH2:22][CH2:21][C:13]3([CH2:20][CH2:19][C:16]4([O:18][CH2:17]4)[CH2:15][CH2:14]3)[C:12]2=[O:23])=[CH:7][CH:6]=1.CC1C=CC(S(O)(=O)=O)=CC=1>C(Cl)(Cl)Cl.C(OCC)(=O)C>[O:23]=[C:12]1[C:13]2([CH2:14][CH2:15][CH:16]([CH:17]=[O:18])[CH2:19][CH2:20]2)[CH2:21][CH2:22][N:11]1[C:8]1[CH:7]=[CH:6][C:5]([O:4][C@H:3]([CH3:24])[C:2]([F:25])([F:1])[F:26])=[CH:10][CH:9]=1

| | **Reaction string dataset (This work)** | **Trust labelling dataset** |
|---|---|---|
| Reactants | 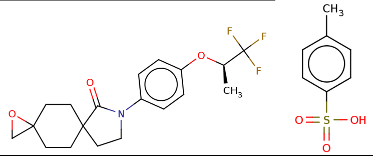 | 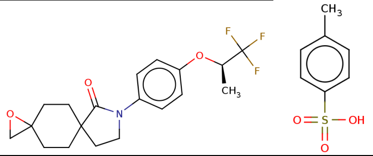 |
| Products | 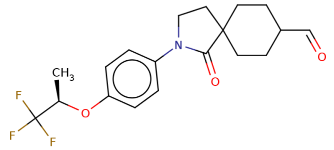 | 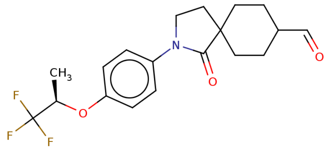 |
| Ground truth solvents | 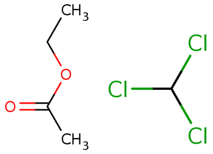 | 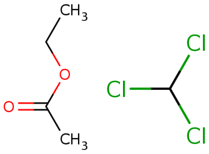 |
| Ground truth agents | None | None |
| Predicted solvents | 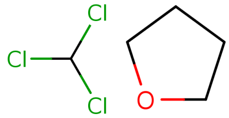 | 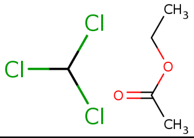 |
| Predicted agents | None | None |

Comment: Correct classification of the reactants. Solvent prediction was correct for the trust labelling dataset, and for the reaction string dataset THF was predicted, which has very similar chemical properties. Agents were marked as None for both datasets.

Correct prediction Incorrect prediction Partially correct prediction

# Datasheet for ORDerly Datasets

## Motivation

Q1: **For what purpose was the dataset created?** Was there a specific task in mind? Was there a specific gap that needed to be filled? Please provide a description.

- The datasets were created to facilitate building machine learning models for prediction of reaction products, retrosynthesis, and reaction conditions in chemical synthesis, particularly in the context of the pharmaceutical industry. There was a need of a clean, high-quality reaction condition benchmark dataset, in addition to a need for an open-source repository for cleaning reactions, and an investigation of how decisions made during cleaning impact the usefulness of the model that is trained on the datasets. ORDerly solves all three of these issues. The code for ORDerly, and the raw data used to generate the ORDerly benchmark datasets, are both open-source, making the benchmark generation accessible and reproducible.

Q2: **Who created the dataset (e.g., which team, research group) and on behalf of which entity (e.g., company, institution, organization)?**

- ORDerly was built by researchers from the group of Professor Alexei Lapkin at the University of Cambridge.

Q3: **Who funded the creation of the dataset?** If there is an associated grant, please provide the name of the grantor and the grant name and number.

- This work is co-funded by UCB Pharma and Engineering and Physical Sciences Research Council via project EP/S024220/1 EPSRC Centre for Doctoral Training in Automated Chemical Synthesis Enabled by Digital Molecular Technologies. This project was co-funded by European Regional Development Fund via the project "Innovation Centre in Digital Molecular Technologies".

Q4: **Any other comments?**

- No.

## Composition

Q5: **What do the instances that comprise the dataset represent (e.g., documents, photos, people, countries)?** *Are there multiple types of instances (e.g., movies, users, and ratings; people and interactions between them; nodes and edges)? Please provide a description.*

- Nine datasets were presented in this work. Each dataset was saved in Apache Parquet format, and has the following column groups:
  - Reaction SMILES string (string), is_mapped (bool)
  - Reactants & products (SMILES strings)
  - Solvents and agents (rxn string data), or solvents, catalysts, and reagents (labeling data) (SMILES strings)
  - Temperature, reaction time, yield (floats)
  - Procedure details (string)
  - Grant date (datetime), date of experiment (datetime), file name (string)

Q6: **How many instances are there in total (of each type, if appropriate)?**

- The number of reactions in each dataset is outlined in detail in Table 1.

Q7: **Does the dataset contain all possible instances or is it a sample (not necessarily random) of instances from a larger set?** *If the dataset is a sample, then what is the larger set? Is the sample representative of the larger set (e.g., geographic coverage)? If so, please describe how this representativeness was validated/verified. If it is not representative of the larger set, please describe why not (e.g., to cover a more diverse range of instances, because instances were withheld or unavailable).*

- All the data in ORD was used to generate the datasets presented in this paper. The ORDerly benchmark datasets were built from the subset of ORD belonging to USPTO (1.7m reactions in total), while the non-USPTO datasets were built on the subset of data from ORD that do not belong to USPTO (94k reactions in total, as of January 2024).

Q8: **What data does each instance consist of?** *"Raw" data (e.g., unprocessed text or images) or features? In either case, please provide a description.*

- Chemical reaction data stored in ORD is structured like a json/dictionary, with strings and floats as the values. The values that are relevant to ORDerly were discussed in response to Q5. A full description of the data stored in ORD is available elsewhere.[18]

Q9: **Is there a label or target associated with each instance?** *If so, please provide a description.*

- There is a label associated with molecules in ORD, and in this work we show the pitfalls of relying on this label, and present ORDerly to more robustly assign labels. The targets are the reaction conditions (solvents, agents, catalysts, reagents).

Q10: **Is any information missing from individual instances?** *If so, please provide a description, explaining why this information is missing (e.g., because it was unavailable). This does not include intentionally removed information, but might include, e.g., redacted text.*

- Many reactions were missing temperature, reaction time, and yield data; this is likely due to this information not being recorded by the experimentalist, or not extracted when the information was scraped from a patent/paper.

Q11: **Are relationships between individual instances made explicit (e.g., users'**

**movie ratings, social network links)?** *If so, please describe how these relationships are made explicit.*

- Each row contains information for a single step chemical reaction. The only explicit link between reactions is the year they were performed or the year that the corresponding patent was granted. The year a chemical reaction was performed may imply some degree of chemical information, since chemical reactions of a certain type obviously could not have been performed before they were invented. Furthermore, "hype" around a particular type of reaction may influence how often certain reaction classes are used through time. For these reasons, a time-based split can be viewed as a (somewhat poor) proxy for a reaction class split. There is a column in the dataset containing the year that the grant was awarded, and another column for time of experiment.

Q12: **Are there recommended data splits (e.g., training, development/validation, testing)?** If so, please provide a description of these splits, explaining the rationale behind them.

- We recommend using a random split of the ORDerly benchmarks, and provide pre-split data to ensure that ML researchers using the benchmark use the same train/test split. There are three data splits that would make sense on a chemical reactions dataset: a random split, a time split, and the reaction class split. A reaction class split would require models to generalize to unseen reaction classes (as opposed to unseen reactions of the same class), making the prediction task much more difficult. As explained above (Q16), using a time split would effectively just serve as a proxy for a reaction class split, and is therefore not desirable. There are a number of reasons for the random split being preferred over the reaction class split: 1) A reaction class split would need to either use an ML clustering algorithm (which usually work quite well, but cannot be viewed as a ground-truth split),

or using proprietary software based on manually curated chemistry rules (which would mean that the full pipeline is no longer fully open source and reproducible). 2) The reaction prediction task is already difficult enough with a random split, and models trained on a random split are still able to provide value even if they can only make predictions on reaction classes that they have seen before - the reaction classes represented in the dataset will likely be the most popular reaction classes, and therefore also those most likely to be queried by the end user.

Q13: **Are there any errors, sources of noise, or redundancies in the dataset?** *If so, please provide a description.*

- The `ORDerly-condition`, `ORDerly-forward`, and `ORDerly-retro` datasets are generated from the USPTO dataset, which is a dataset made from chemical reactions from US Patents. When a molecule is patented, it is also a requirement to publish the synthesis pathway to produce the molecule, and it is from these synthesis pathways that reactions are extracted. To avoid giving away proprietary information there is an incentive to use already published "industry standard" reaction conditions in the patent application; furthermore, the "first to file" nature of the US patent system means there is an incentive to apply for patents as soon as possible. These two factors may bias the reactions in the USPTO dataset towards being unoptimized, low-yielding reactions that can also be found elsewhere. In fact, we observed that $\approx 40\%$ of reactions were dropped because they were duplicates (see Table 1), indicating that many reactions are executed at "standard conditions" for a particular class of reaction instead of being optimized for the specific reactants.
- Reproducibility is known to be difficult in chemistry,[19] which implies a base-level of noise in the dataset.

Q14: **Is the dataset self-contained, or does it link to or otherwise rely on external**

**resources (e.g., websites, tweets, other datasets)?** *If it links to or relies on external resources, a) are there guarantees that they will exist, and remain constant, over time; b) are there official archival versions of the complete dataset (i.e., including the external resources as they existed at the time the dataset was created); c) are there any restrictions (e.g., licenses, fees) associated with any of the external resources that might apply to a future user? Please provide descriptions of all external resources and any restrictions associated with them, as well as links or other access points, as appropriate.*

- The ORDerly datasets are self-contained. To be able to reproduce cleaning of ORD data, the ORD data will naturally need to continue to exist. ORD was built to be an open-source tool, so there should not be any restrictions on its use in the future.

Q15: **Does the dataset contain data that might be considered confidential (e.g., data that is protected by legal privilege or by doctor–patient confidentiality, data that includes the content of individuals' non-public communications)?** *If so, please provide a description.*

- No.

Q16: **Does the dataset contain data that, if viewed directly, might be offensive, insulting, threatening, or might otherwise cause anxiety?** *If so, please describe why.*

- No.

Q17: **Does the dataset relate to people?** *If not, you may skip the remaining questions in this section.*

- No.

Q18: **Does the dataset identify any subpopulations (e.g., by age, gender)?**

- No.

Q19: **Is it possible to identify individuals (i.e., one or more natural persons), either directly or indirectly (i.e., in combination with other data) from the dataset?** *If so, please describe how.*

- No.

Q20: **Does the dataset contain data that might be considered sensitive in any way (e.g., data that reveals racial or ethnic origins, sexual orientations, religious beliefs, political opinions or union memberships, or locations; financial or health data; biometric or genetic data; forms of government identification, such as social security numbers; criminal history)?** *If so, please provide a description.*

- No.

Q21: **Any other comments?**

- No.

## Collection Process

Q22: **How was the data associated with each instance acquired?** Was the data directly observable (e.g., raw text, movie ratings), reported by subjects (e.g., survey responses), or indirectly inferred/derived from other data (e.g., part-of-speech tags, model-based guesses for age or language)? If data was reported by subjects or indirectly inferred/derived from other data, was the data validated/verified? If so, please describe how.

- The raw data of each instance (reaction) was extracted from United States Patents to form the "USPTO dataset".[20] The USPTO dataset was parsed into ORD

format,[18] where we extracted it from. ORD does contain additional data, beyond the USPTO dataset. Other reactions in ORD are contributed by chemists in academia and industry.

Q23: **What mechanisms or procedures were used to collect the data (e.g., hardware apparatus or sensor, manual human curation, software program, software API)?** How were these mechanisms or procedures validated?

- Data in the ORD database is readily downloadable through the GitHub repository: `https://github.com/open-reaction-database/ord-data`.

Q24: **If the dataset is a sample from a larger set, what was the sampling strategy (e.g., deterministic, probabilistic with specific sampling probabilities)?**

- See Q7.

Q25: **Who was involved in the data collection process (e.g., students, crowdworkers, contractors) and how were they compensated (e.g., how much were crowdworkers paid)?**

- N/A.

Q26: **Over what timeframe was the data collected? Does this timeframe match the creation timeframe of the data associated with the instances (e.g., recent crawl of old news articles)?** *If not, please describe the timeframe in which the data associated with the instances was created.*

- The reactions in the USPTO dataset are from patents which were published between 1976 and September 2016. The USPTO dataset was parsed into ORD in 2020. Additional reactions not from patents have since been added to ORD. ORDerly was built in 2023.

Q27: **Were any ethical review processes conducted (e.g., by an institutional review board)?** *If so, please provide a description of these review processes, including the outcomes, as well as a link or other access point to any supporting documentation.*

- No.

Q28: **Does the dataset relate to people?** *If not, you may skip the remaining questions in this section.*

- No.

Q29: **Did you collect the data from the individuals in question directly, or obtain it via third parties or other sources (e.g., websites)?**

- N/A.

Q30: **Were the individuals in question notified about the data collection?** *If so, please describe (or show with screenshots or other information) how notice was provided, and provide a link or other access point to, or otherwise reproduce, the exact language of the notification itself.*

- N/A.

Q31: **Did the individuals in question consent to the collection and use of their data?** *If so, please describe (or show with screenshots or other information) how consent was requested and provided, and provide a link or other access point to, or otherwise reproduce, the exact language to which the individuals consented.*

- N/A.

Q32: **If consent was obtained, were the consenting individuals provided with a mechanism to revoke their consent in the future or for certain uses?** *If so, please provide a description, as well as a link or other access point to the mechanism (if appropriate).*

- N/A.

Q33: **Has an analysis of the potential impact of the dataset and its use on data subjects (e.g., a data protection impact analysis) been conducted?** *If so, please provide a description of this analysis, including the outcomes, as well as a link or other access point to any supporting documentation.*

- N/A.

Q34: **Any other comments?**

- No.

## Preprocessing, Cleaning, and/or Labeling

Q35: **Was any preprocessing/cleaning/labeling of the data done (e.g., discretization or bucketing, tokenization, part-of-speech tagging, SIFT feature extraction, removal of instances, processing of missing values)?** If so, please provide a description. If not, you may skip the remainder of the questions in this section.

- Yes, this is described in detail in the Methodology section of the paper and the Dataset Extraction and Cleaning section in the SI.

Q36: **Was the "raw" data saved in addition to the preprocessed/cleaned/labeled data (e.g., to support unanticipated future uses)?** If so, please provide a link or other access point to the "raw" data.

- The raw structured data is stored in the ORD GitHub repository: `https://github.com/open-reaction-database/ord-data`.

Q37: **Is the software used to preprocess/clean/label the instances available?** If so, please provide a link or other access point.

- This paper is for the software used to preprocess, clean, and label the instances.

Q38: **Any other comments?**

- No.

## Uses

Q39: **Has the dataset been used for any tasks already?** *If so, please provide a description.*

- Yes, we train a previously published neural network model for reaction condition prediction and a previously published transformer for forward prediction and retrosynthesis.

Q40: **Is there a repository that links to any or all papers or systems that use the dataset?** If so, please provide a link or other access point.

- No.

Q41: **What (other) tasks could the dataset be used for?**

- Key problems in chemical synthesis include reaction outcome prediction, retrosynthesis, and reaction condition prediction, all described in this work. Another important task, which was not described here, is reaction yield prediction. Successful reaction yield models are predominantly trained on high-throughput experimentation (HTE) datasets,[5] and is known to be difficult (if not impossible) with patent data (e.g. USPTO).[21,22] As long as ORD primarily consists of USPTO data, ORDerly will probably not be very useful for yield prediction, but it could be in the future.

Q42: **Is there anything about the composition of the dataset or the way it was collected and preprocessed/cleaned/labeled that might impact future uses?**

*For example, is there anything that a future user might need to know to avoid uses that could result in unfair treatment of individuals or groups (e.g., stereotyping, quality of service issues) or other undesirable harms (e.g., financial harms, legal risks) If so, please provide a description. Is there anything a future user could do to mitigate these undesirable harms?*

- Yes, ORDerly relies on the ORD schema, and changes to the ORD schema or ORD database may require updates to ORDerly. ORD may change in the future, as the it becomes more clear how the community wishes to use ORD (e.g. which classes of information are stored).

Q43: **Are there tasks for which the dataset should not be used?** *If so, please provide a description.*

- The ORDerly datasets were generated to make it easier to train models that can predict how to make small molecules. The intended usage is to predict synthesis pathways for therapeutics, however, within this category of small molecules is also energetic materials, such as explosives.

Q44: **Any other comments?**

- No.

## Distribution

Q45: **Will the dataset be distributed to third parties outside of the entity (e.g., company, institution, organization) on behalf of which the dataset was created?** If so, please provide a description.

- Yes, the datasets will be open-source.

Q46: **How will the dataset be distributed (e.g., tarball on website, API, GitHub)?** Does the dataset have a digital object identifier (DOI)?

- The data is available through FigShare: `https://doi.org/10.6084/m9.figshare.23298467`
- It can also reliably be recreated using the instructions in the ORDerly GitHub repository `https://github.com/sustainable-processes/ORDerly`

Q47: **When will the dataset be distributed?**

- It is already publicly available.

Q48: **Will the dataset be distributed under a copyright or other intellectual property (IP) license, and/or under applicable terms of use (ToU)?** *If so, please describe this license and/or ToU, and provide a link or other access point to, or otherwise reproduce, any relevant licensing terms or ToU, as well as any fees associated with these restrictions.*

- CC-BY-4.0

Q49: **Have any third parties imposed IP-based or other restrictions on the data associated with the instances?** *If so, please describe these restrictions, and provide a link or other access point to, or otherwise reproduce, any relevant licensing terms, as well as any fees associated with these restrictions.*

- No.

Q50: **Do any export controls or other regulatory restrictions apply to the dataset or to individual instances?** *If so, please describe these restrictions, and provide a link or other access point to, or otherwise reproduce, any supporting documentation.*

- No,

Q51: **Any other comments?**

- No.

## Maintenance

Q52: **Who will be supporting/hosting/maintaining the dataset?**

- The dataset is hosted on FigShare, the code to generate the dataset is hosted on GitHub.
- The group of Prof Alexei Lapkin will be maintaining ORDerly.

Q53: **How can the owner/curator/manager of the dataset be contacted (e.g., email address)?**

- Prof Alexei Lapkin, the corresponding author, can be contacted at aal35@cam.ac.uk.

Q54: **Is there an erratum?** If so, please provide a link or other access point.

- N/A.

Q55: **Will the dataset be updated (e.g., to correct labeling errors, add new instances, delete instances)?** *If so, please describe how often, by whom, and how updates will be communicated to users (e.g., mailing list, GitHub)?*

- ORDerly will be maintained by the group of Prof Alexei Lapkin, updates will be tracked through GitHub. ORDerly is built to be extensible, such that as the ORD dataset grows, users can run ORDerly to create new, larger, datasets. The ORDerly benchmark datasets are unlikely to change (to ensure model accuracy is comparable).

Q56: **If the dataset relates to people, are there applicable limits on the retention of the data associated with the instances (e.g., were individuals in question**

**told that their data would be retained for a fixed period of time and then deleted)?** *If so, please describe these limits and explain how they will be enforced.*

- N/A.

Q57: **Will older versions of the dataset continue to be supported/hosted/maintained?** If so, please describe how. If not, please describe how its obsolescence will be communicated to users.

- The datasets are small enough to easily be versioned and hosted on FigShare (350k-1m reactions, 200MB-500MB).

Q58: **If others want to extend/augment/build on/contribute to the dataset, is there a mechanism for them to do so?** *If so, please provide a description. Will these contributions be validated/verified? If so, please describe how. If not, why not? Is there a process for communicating/distributing these contributions to other users? If so, please provide a description*

- All contributions to ORDerly will be managed through the ORDerly GitHub repository. Pull requests into main will need to be verified by a member of Prof Alexei Lapkin's group.

Q59: **Any other comments?**

- No.

# References

(1) Meng, Z.; Zhao, P.; Yu, Y.; King, I. A Unified View of Deep Learning for Reaction and Retrosynthesis Prediction: Current Status and Future Challenges. Electronic proceedings of IJCAI 2023. 2023; pp 6723–6731.

(2) Wigh, D. S.; Goodman, J. M.; Lapkin, A. A. A review of molecular representation in the age of machine learning. *WIREs Comput. Mol. Sci.* **2022**, *12*, e1603.

(3) Coley, C. W. et al. A robotic platform for flow synthesis of organic compounds informed by AI planning. *Science* **2019**, *365*.

(4) Gao, H.; Struble, T. J.; Coley, C. W.; Wang, Y.; Green, W. H.; Jensen, K. F. Using Machine Learning To Predict Suitable Conditions for Organic Reactions. *ACS Cent. Sci.* **2018**, *4*, 1465–1476.

(5) Schwaller, P.; Vaucher, A. C.; Laino, T.; Reymond, J.-L. Prediction of chemical reaction yields using deep learning. *Mach. Learn.: Sci. Technol.* **2021**, *2*, 015016.

(6) Neves, P.; McClure, K.; Verhoeven, J.; Dyubankova, N.; Nugmanov, R.; Gedich, A.; Menon, S.; Shi, Z.; Wegner, J. K. Global reactivity models are impactful in industrial synthesis applications. *J. Cheminf.* **2023**, *15*, 20.

(7) Amar, Y.; Schweidtmann, A. M.; Deutsch, P.; Cao, L.; Lapkin, A. Machine learning and molecular descriptors enable rational solvent selection in asymmetric catalysis. *Chem. Sci.* **2019**, *10*, 6697–6706.

(8) Diorazio, L. J.; Hose, D. R. J.; Adlington, N. K. Toward a More Holistic Framework for Solvent Selection. *Organic Process Research & Development* **2016**, *20*, 760–773.

(9) Felton, K. C.; Rittig, J. G.; Lapkin, A. A. Summit: Benchmarking Machine Learning Methods for Reaction Optimisation. *Chemistry–Methods* **2021**, *1*, 116–122.

(10) Felton, K. Pura. 2023; `https://github.com/sustainable-processes/pura`.

(11) Arun, A.; Guo, Z.; Sung, S.; Lapkin, A. A. Reaction Impurity Prediction using a Data Mining Approach**. *Chemistry–Methods* **2023**,

(12) Schneider, N.; Stiefl, N.; Landrum, G. A. What's What: The (Nearly) Definitive Guide to Reaction Role Assignment. *JCIM* **2016**, *56*, 2336–2346.

(13) Liu, B.; Ramsundar, B.; Kawthekar, P.; Shi, J.; Gomes, J.; Luu Nguyen, Q.; Ho, S.; Sloane, J.; Wender, P.; Pande, V. Retrosynthetic Reaction Prediction Using Neural Sequence-to-Sequence Models. *ACS central science* **2017**, *3*, 1103–1113.

(14) Coley, C. W.; Rogers, L.; Green, W. H.; Jensen, K. F. Computer-Assisted Retrosynthesis Based on Molecular Similarity. *ACS Cent. Sci.* **2017**, *3*, 1237–1245.

(15) Jin, W.; Coley, C.; Barzilay, R.; Jaakkola, T. Predicting Organic Reaction Outcomes with Weisfeiler-Lehman Network. Advances in Neural Information Processing Systems. 2017.

(16) Huang, K.; Fu, T.; Gao, W.; Zhao, Y.; Roohani, Y.; Leskovec, J.; Coley, C.; Xiao, C.; Sun, J.; Zitnik, M. Therapeutics Data Commons: Machine Learning Datasets and Tasks for Drug Discovery and Development. *Proceedings of the Neural Information Processing Systems Track on Datasets and Benchmarks* **2021**, *1*.

(17) Schwaller, P.; Laino, T.; Gaudin, T.; Bolgar, P.; Hunter, C. A.; Bekas, C.; Lee, A. A. Molecular Transformer: A Model for Uncertainty-Calibrated Chemical Reaction Prediction. *ACS Cent. Sci.* **2019**, *5*, 1572–1583.

(18) Kearnes, S. M.; Maser, M. R.; Wleklinski, M.; Kast, A.; Doyle, A. G.; Dreher, S. D.; Hawkins, J. M.; Jensen, K. F.; Coley, C. W. The Open Reaction Database. *JACS* **2021**, *143*, 18820–18826.

(19) Baker, M. 1,500 scientists lift the lid on reproducibility. *Nature* **2016**, *533*, 452–454.

(20) Lowe, D. Chemical reactions from US patents (1976-Sep2016). 2017; `https://figshare.com/articles/Chemical_reactions_from_US_patents_1976-Sep2016_/5104873`.

(21) Probst, D.; Schwaller, P.; Reymond, J.-L. Reaction classification and yield prediction using the differential reaction fingerprint DRFP. *Digital Discovery* **2022**, *1*, 91–97.

(22) Fitzner, M.; Wuitschik, G.; Koller, R.; Adam, J.-M.; Schindler, T. Machine Learning C–N Couplings: Obstacles for a General-Purpose Reaction Yield Prediction. *ACS Omega* **2023**, *8*, 3017–3025.
